# Supplementary material for: Cavity frequency-dependent theory for vibrational polariton chemistry
Source: Nat Commun. 2021 Feb 26;12:1315. doi: 10.1038/s41467-021-21610-9 (PMC7910560; doi:10.1038/s41467-021-21610-9)
Supplement: Supplementary file 1 — Supplementary Information [file 41467_2021_21610_MOESM1_ESM.pdf]

Supplementary Information for Cavity Frequency-Dependent Theory for Vibrational Polariton Chemistry

Xinyang Li, Arkajit Mandal,* and Pengfei Huo*

Department of Chemistry, University of Rochester, 120 Trustee Road, Rochester, New York 14627, United States

E-mail: amandal4@ur.rochester.edu; pengfei.huo@rochester.edu

Supplementary Note 1. Derivation of the Pauli-Fierz QED Hamiltonian

We provide a brief derivation of the Pauli-Fierz QED Hamiltonian. We begin by defining the matter Hamiltonian and the corresponding total dipole operator as follows

$$\hat{H}_{\mathrm{M}} = \hat{\mathbf{T}} + \hat{V}(\hat{\mathbf{x}}) = \sum_j \frac{1}{2m_j}\hat{\mathbf{p}}_j^2 + \hat{V}(\hat{\mathbf{x}});\ \hat{\boldsymbol{\mu}} = \sum_j z_j \hat{\mathbf{x}}_j, \tag{1}$$

where j is the index of the j_{th} charged particle (including all electrons and nuclei), with the corresponding mass m_j and charge z_j. In addition, $\hat{\mathbf{x}} \equiv \{\hat{\mathbf{x}}_j\} = \{\hat{\mathbf{R}}, \hat{\mathbf{r}}\}$ with $\hat{\mathbf{R}}$ and $\hat{\mathbf{r}}$ representing the nuclear and electronic coordinates, respectively, $\hat{\mathbf{p}} \equiv \{\hat{\mathbf{p}}_{\mathbf{R}}, \hat{\mathbf{p}}_{\mathbf{r}}\} \equiv \{\hat{\mathbf{p}}_j\}$ is the *mechanical* momentum operator as well as the canonical momentum operator, such that $\hat{\mathbf{p}}_j = -i\hbar\boldsymbol{\nabla}_j$. Further, $\hat{\mathbf{T}} = \hat{\mathbf{T}}_{\mathbf{R}} + \hat{\mathbf{T}}_{\mathbf{r}}$ is the kinetic energy operator, where $\hat{\mathbf{T}}_{\mathbf{R}}$ and $\hat{\mathbf{T}}_{\mathbf{r}}$ represent the kinetic energy operator for nuclei and for electrons, respectively, and $\hat{V}(\hat{\mathbf{x}})$ is the potential operator that describes the Coulombic interactions among electrons and nuclei.

The cavity photon field Hamiltonian under the single-mode assumption is expressed as

$$\hat{H}_{\mathrm{ph}} = \hbar\omega_{\mathrm{c}}\big(\hat{a}^{\dagger}\hat{a} + \frac{1}{2}\big) = \frac{1}{2}\big(\hat{p}_{\mathrm{c}}^2 + \omega_{\mathrm{c}}^2\hat{q}_{\mathrm{c}}^2\big), \tag{2}$$

where ω_{c} is the frequency of the mode in the cavity, $\hat{a}^{\dagger}$ and $\hat{a}$ are the photonic creation and annihilation operators, and $\hat{q}_{\mathrm{c}} = \sqrt{\hbar/2\omega_{\mathrm{c}}}(\hat{a}^{\dagger} + \hat{a})$ and $\hat{p}_{\mathrm{c}} = i\sqrt{\hbar\omega_{\mathrm{c}}/2}(\hat{a}^{\dagger} - \hat{a})$ are the photonic

coordinate and momentum operators, respectively. Choosing the Coulomb gauge, $\boldsymbol{\nabla}\cdot\hat{\mathbf{A}}=0$, the vector potential becomes purely transverse $\hat{\mathbf{A}}=\hat{\mathbf{A}}_{\perp}$. Under the long-wavelength approximation,

$$\hat{\mathbf{A}}=\mathbf{A}_0\big(\hat{a}+\hat{a}^\dagger\big)=\mathbf{A}_0\sqrt{2\omega_{\mathrm{c}}/\hbar}\;\hat{q}_{\mathrm{c}}, \tag{3}$$

where $\mathbf{A}_0=\sqrt{\hbar/2\omega_{\mathrm{c}}\varepsilon_0\mathcal{V}}\;\hat{\mathbf{e}}$, with $\mathcal{V}$ as the quantization volume inside the cavity, ε_0 as the permittivity, and $\hat{\mathbf{e}}$ is the unit vector of the field polarization.

We further introduce the Power-Zienau-Woolley (PZW) gauge transformation operator[1,2] as

$$\hat{U}=\exp\big[-\frac{i}{\hbar}\hat{\boldsymbol{\mu}}\cdot\hat{\mathbf{A}}\big]=\exp\big[-\frac{i}{\hbar}\hat{\boldsymbol{\mu}}\cdot\mathbf{A}_0\big(\hat{a}+\hat{a}^\dagger\big)\big]. \tag{4}$$

The PZW transformation operator can also be expressed as $\hat{U}=\exp\big[-\frac{i}{\hbar}\sqrt{2\omega_{\mathrm{c}}/\hbar}\hat{\boldsymbol{\mu}}\mathbf{A}_0\hat{q}_{\mathrm{c}}\big]=\exp\big[-\frac{i}{\hbar}(\sum_j z_j\hat{\mathbf{A}}\mathbf{x}_j)\big]$. Recall that a momentum boost operator $\hat{U}_{\mathrm{p}}=e^{-\frac{i}{\hbar}p_0\hat{q}}$ displaces $\hat{p}$ by the amount of p_0, such that $\hat{U}_{\mathrm{p}}\hat{O}(\hat{p})\hat{U}_{\mathrm{p}}^\dagger=\hat{O}(\hat{p}+p_0)$. Hence, $\hat{U}$ is a boost operator for both the photonic momentum $\hat{p}_{\mathrm{c}}$ by the amount of $\sqrt{2\omega_{\mathrm{c}}/\hbar}\hat{\boldsymbol{\mu}}\mathbf{A}_0$, as well as for the matter momentum $\hat{\mathbf{p}}_j$ by the amount of $z_j\hat{\mathbf{A}}$. Using $\hat{U}^\dagger$ to boost the matter momentum, one can show that

$$\hat{H}_{\mathrm{C}}=\hat{U}^\dagger\hat{H}_{\mathrm{M}}\hat{U}+\hat{H}_{\mathrm{ph}}, \tag{5}$$

hence $\hat{H}_{\mathrm{C}}$ can be obtained[3] by a momentum boost with the amount of $-z_j\hat{\mathbf{A}}$ for $\hat{\mathbf{p}}_j$, then adding $\hat{H}_{\mathrm{ph}}$.

The QED Hamiltonian under the *dipole* gauge (the "d · E" form[1,4]) can be obtained by performing the PZW transformation on $\hat{H}_{\mathrm{C}}$ as follows

$$\hat{H}_{\mathrm{D}}=\hat{U}\hat{H}_{\mathrm{C}}\hat{U}^\dagger=\hat{U}\hat{U}^\dagger\hat{H}_{\mathrm{M}}\hat{U}\hat{U}^\dagger+\hat{U}\hat{H}_{\mathrm{ph}}\hat{U}^\dagger=\hat{H}_{\mathrm{M}}+\hbar\omega_{\mathrm{c}}(\hat{a}^\dagger\hat{a}+\frac{1}{2})+i\omega_{\mathrm{c}}\hat{\boldsymbol{\mu}}\mathbf{A}_0(\hat{a}^\dagger-\hat{a})+\frac{\omega_{\mathrm{c}}}{\hbar}(\hat{\boldsymbol{\mu}}\mathbf{A}_0)^2, \tag{6}$$

where we have used Supplementary Eq. 5 to express $\hat{H}_{\mathrm{C}}$, and the last three terms of the above equation are the results of $\hat{U}\hat{H}_{\mathrm{ph}}\hat{U}^\dagger$. Using $\hat{q}_{\mathrm{c}}$ and $\hat{p}_{\mathrm{c}}$, one can instead show that

$$\hat{H}_{\mathrm{D}}=\hat{H}_{\mathrm{M}}+\frac{1}{2}\omega_{\mathrm{c}}^2\hat{q}_{\mathrm{c}}^2+\frac{1}{2}(\hat{p}_{\mathrm{c}}+\sqrt{2\omega_{\mathrm{c}}/\hbar}\hat{\boldsymbol{\mu}}\mathbf{A}_0)^2, \tag{7}$$

because the PZW operator boosts the photonic momentum $\hat{p}_{\rm c}$ by $\sqrt{2\omega_{\rm c}/\hbar}\hat{\boldsymbol{\mu}}\mathbf{A}_0$. The term $\frac{\omega_{\rm c}}{\hbar}(\hat{\boldsymbol{\mu}}\mathbf{A}_0)^2$ is commonly referred to as the dipole self-energy (DSE).

The Pauli-Fierz (PF) QED Hamiltonian[5–7] can be obtained by using a unitary transformation $\hat{U}_\phi = \exp\big[i\frac{\pi}{2}\hat{a}^\dagger\hat{a}\big]$ on $\hat{H}_{\rm D}$. To proceed, we use the following Baker-Campbell-Hausdorff (BCH) identity

$$e^{\hat{A}}\hat{B}e^{-\hat{A}} = \hat{B} + [\hat{A},\hat{B}] + \frac{1}{2!}[\hat{A},[\hat{A},\hat{B}]] + ... \tag{8}$$

Using the fundamental commutator $[\hat{a}^\dagger,\hat{a}] = -1$, we have $[\hat{a}^\dagger\hat{a},\hat{a}] = \hat{a}^\dagger[\hat{a},\hat{a}] + [\hat{a}^\dagger,\hat{a}]\hat{a} = -\hat{a}$. Denoting $\hat{U}_\phi = \exp\big[i\phi\hat{a}^\dagger\hat{a}\big] = e^{-\hat{A}}$ (with $\phi = \frac{\pi}{2}$), hence $\hat{A} = -i\phi\hat{a}^\dagger\hat{a}$. Using the BCH identity, we have

$$\begin{aligned} e^{-i\phi\hat{a}^\dagger\hat{a}}\hat{a}e^{i\phi\hat{a}^\dagger\hat{a}} &= \hat{a} - i\phi[\hat{a}^\dagger\hat{a},\hat{a}] + \frac{1}{2!}(-i\phi)^2[\hat{a}^\dagger\hat{a},[\hat{a}^\dagger\hat{a},\hat{a}]] + ... \\ &= \Big(1 + (-i\phi)(-1) + \frac{1}{2!}(-i\phi)^2(-1)^2 + ...\Big)\hat{a} \\ &= e^{i\phi}\hat{a} \end{aligned} \tag{9}$$

Similarly, we have $e^{-i\phi\hat{a}^\dagger\hat{a}}\hat{a}^\dagger e^{i\phi\hat{a}^\dagger\hat{a}} = e^{-i\phi}\hat{a}^\dagger$. Choosing $\phi = \frac{\pi}{2}$ results in $\hat{U}_\phi^\dagger\hat{a}\hat{U}_\phi \to i\hat{a}$ and $\hat{U}_\phi^\dagger\hat{a}^\dagger\hat{U}_\phi \to -i\hat{a}^\dagger$. Using these results, and applying $\hat{U}_\phi$ on $\hat{H}_{\rm D}$, we have the PF Hamiltonian as follows

$$\hat{H}_{\rm PF} = \hat{U}_\phi\hat{H}_{\rm D}\hat{U}_\phi^\dagger = \hat{H}_{\rm M} + \hbar\omega_{\rm c}(\hat{a}^\dagger\hat{a} + \frac{1}{2}) + \mathbf{A}_0\omega_{\rm c}\hat{\boldsymbol{\mu}}(\hat{a} + \hat{a}^\dagger) + \frac{\omega_{\rm c}}{\hbar}(\mathbf{A}_0\hat{\boldsymbol{\mu}})^2 = \hat{H}_{\rm M} + \frac{1}{2}\hat{p}_{\rm c}^2 + \frac{1}{2}\omega_{\rm c}^2(\hat{q}_{\rm c} + \frac{A_0\hat{\boldsymbol{\mu}}}{\sqrt{\hbar\omega_{\rm c}}})^2. \tag{10}$$

Note that we have used the fact that $\hat{U}_\phi\hat{H}_{\rm M}\hat{U}_\phi^\dagger = \hat{H}_{\rm M}$, *i.e.*, $\hat{U}_\phi$ does not contain any matter DOFs. Hence, the role of $\hat{U}_\phi$ is to switch $\hat{p}_{\rm c}$ and $\hat{q}_{\rm c}$, and for a photon field, they are inter-changeable due to the pure harmonic nature of the quantized field. The PF Hamiltonian has the advantage as a pure real Hamiltonian and the photonic DOF can be viewed[5,7] and computationally treated[8,9] as "nuclear coordinates". Projecting the above Hamiltonian in the ground electronic state of the molecule $|\Psi_g\rangle$ (which is obtained by solving $\hat{H}_{\rm el}|\Psi_g\rangle = E(R)|\Psi_g\rangle$, we obtain the model Hamiltonian, that is $\hat{H}_{\rm PF} = \hat{T}_{\rm R} + E(R) + \hat{H}_{\rm vib} + \frac{1}{2}\hat{p}_{\rm c}^2 + \frac{1}{2}\omega_{\rm c}^2(\hat{q}_{\rm c} + \frac{A_0\mu(R)}{\sqrt{\hbar\omega_{\rm c}}})^2$, in Eq. 1 of the main text, which is depicted in Fig. 1b of the main text.

Supplementary Note 2. Details of the Normal Mode Analysis.

Here we provide the detailed derivations of the normal mode frequencies used in the GH rate theory. The polariton Hamiltonian is defined as $\hat{H}_{\rm pl} = \hat{H} - \frac{\hat{P}^2}{2M} - \hat{H}_{\rm vib}$ with the following expression

$$\hat{H}_{\rm pl} = E(R) + \frac{\hat{p}_{\rm c}^2}{2} + \frac{1}{2}\omega_{\rm c}^2\big(\hat{q}_{\rm c} + \sqrt{\frac{2}{\hbar\omega_{\rm c}^3}}\chi\cdot\mu(R)\big)^2. \tag{11}$$

We further view R and q_c as classical coordinates

$$H_{\rm pl}(R, q_{\rm c}) = E(R) + \frac{1}{2}\omega_{\rm c}^2\big(q_{\rm c} + \sqrt{\frac{2}{\hbar\omega_{\rm c}^3}}\chi\cdot\mu(R)\big)^2 = E(R) + \frac{1}{2}\omega_{\rm c}^2 q_{\rm c} + \sqrt{\frac{2\omega_{\rm c}}{\hbar}}\chi\cdot q_{\rm c}\mu(R) + \frac{\chi^2\mu^2(R)}{\hbar\omega_{\rm c}} \tag{12}$$

where R is the nuclear reaction coordinate and $q_{\rm c}$ is the photon mode coordinate. The mass-weighted Hessian matrix $\mathcal{H}$ of this model is defined as

$$\mathcal{H}(R, q_{\rm c}) = \begin{pmatrix} \frac{1}{M}\frac{\partial^2 H_{\rm pl}}{\partial R^2} & \frac{1}{\sqrt{M}}\frac{\partial^2 H_{\rm pl}}{\partial R\partial q_{\rm c}} \\ \frac{1}{\sqrt{M}}\frac{\partial^2 H_{\rm pl}}{\partial q_{\rm c}\partial R} & \frac{\partial^2 H_{\rm pl}}{\partial q_{\rm c}^2} \end{pmatrix} \tag{13}$$

where $M = 1836$ a.u. for the nuclear reaction coordinate (proton coordinate).

The equilibrium point (R_0, q_0) at the reactant side of $H_{\rm pl}$ is located at

$$\frac{\partial H_{\rm pl}}{\partial R}\bigg|_{R_0,q_0} = \frac{\partial E(R)}{\partial R}\bigg|_{R_0} + \omega_{\rm c}^2\Big(q_0 + \sqrt{\frac{2}{\hbar\omega_{\rm c}^3}}\chi\cdot\mu(R_0)\Big)\,\frac{\partial\mu(R)}{\partial R}\bigg|_{R_0} = 0 \tag{14}$$

$$\frac{\partial H_{\rm pl}}{\partial q_{\rm c}}\bigg|_{R_0,q_0} = \omega_{\rm c}^2\Big(q_0 + \sqrt{\frac{2}{\hbar\omega_{\rm c}^3}}\chi\cdot\mu(R_0)\Big) = 0. \tag{15}$$

From Supplementary Eq. 15, we have $q_0 = -\sqrt{\frac{2}{\hbar\omega_{\rm c}^3}}\chi\mu(R_0)$, plugging this back to Supplementary Eq. 14, we have $\frac{\partial H_{\rm pl}}{\partial R}\Big|_{R_0,q_0} = \frac{\partial E(R)}{\partial R}\Big|_{R_0} = 0$, *i.e.*, $H_{\rm pl}$ and $E(R)$ share the same equilibrium position along the R direction. At R_0, the curvature of the reactant well is $\frac{\partial E^2(R)}{\partial R^2}\Big|_{R_0} = M\omega_0^2$, and ω_0 is the bottom of the well frequency of the reactant. We further denote the molecular dipole and its derivative at R_0 as $\mu_0 \equiv \mu(R_0)$ and $\mu_0' \equiv \frac{\partial\mu(R)}{\partial R}\Big|_{R_0}$, respectively. At $R = R_0$ and $q_{\rm c} = q_0$, the

Hessian matrix element $\frac{\partial^2 \hat{H}_{\mathrm{pl}}}{\partial R^2}$ is evaluated as

$$\begin{aligned}\frac{\partial^2 \hat{H}_{\mathrm{pl}}}{\partial R^2}\bigg|_{R_0,q_0} &= \frac{\partial E^2(R)}{\partial R^2}\bigg|_{R_0} + \sqrt{\frac{2\omega_c}{\hbar}}\chi q_{\mathrm{c}}\,\frac{\partial \mu^2(R)}{\partial R^2}\bigg|_{R_0,q_0} + \frac{2\chi^2}{\hbar\omega_{\mathrm{c}}}\left[\mu(R)\,\frac{\partial \mu^2(R)}{\partial R^2}\bigg|_{R_0} + \left(\frac{\partial \mu(R)}{\partial R}\bigg|_{R_0}\right)^2\right] \\ &= \frac{\partial^2 \hat{H}_{\mathrm{pl}}}{\partial R^2} = M\omega_0^2 + \frac{2\chi^2}{\hbar\omega_c}{\mu_0'}^2 \end{aligned} \tag{16}$$

The other terms are also straight-forward to evaluate, resulting in

$$\mathcal{H}_0 = \mathcal{H}(R_0, q_0) = \begin{pmatrix} \omega_0^2 + \frac{\mathcal{C}_0^2}{\omega_{\mathrm{c}}^2} & \mathcal{C}_0 \\ \mathcal{C}_0 & \omega_{\mathrm{c}}^2 \end{pmatrix}, \tag{17}$$

where $\mathcal{C}_0 = \sqrt{\frac{2\omega_{\mathrm{c}}}{M\hbar}}\chi \cdot \mu_0'$. Note that making an approximation[10] of the dipole operator as $\mu(R) \approx \mu_0 + \mu_0'(R - R_0)$ in H_{pl} gives the same results of $\mathcal{H}$, although it is not necessary to make such an approximation.

The normal mode frequencies $\Omega_\pm$ are obtained by diagonalizing Supplementary Eq. 17, resulting in

$$\begin{aligned}\Omega_\pm^2 &= \frac{1}{2}(\omega_0^2 + \frac{\mathcal{C}_0^2}{\omega_{\mathrm{c}}^2} + \omega_{\mathrm{c}}^2) \pm \frac{1}{2}\sqrt{(\omega_0^2 + \frac{\mathcal{C}_0^2}{\omega_{\mathrm{c}}^2} + \omega_{\mathrm{c}}^2)^2 - 4\omega_{\mathrm{c}}^2\omega_0^2} \\ &= \frac{1}{2}(\omega_0^2 + \frac{\mathcal{C}_0^2}{\omega_{\mathrm{c}}^2} + \omega_{\mathrm{c}}^2) \pm \frac{1}{2}\sqrt{(\omega_0^2 + \frac{\mathcal{C}_0^2}{\omega_{\mathrm{c}}^2} - \omega_{\mathrm{c}}^2)^2 + 4\mathcal{C}_0^2}.\end{aligned} \tag{18}$$

Similarly, the saddle point $(R_\ddagger, q_\ddagger)$ of H_{pl} is located at

$$\frac{\partial H_{\mathrm{pl}}}{\partial R}\bigg|_{R_\ddagger,q_\ddagger} = \frac{\partial E(R)}{\partial R}\bigg|_{R_\ddagger} + \omega_{\mathrm{c}}^2\Big(q_\ddagger + \sqrt{\frac{2}{\hbar\omega_{\mathrm{c}}^3}}\chi \cdot \mu(R_\ddagger)\Big)\,\frac{\partial \mu(R)}{\partial R}\bigg|_{R_\ddagger} = 0 \tag{19}$$

$$\frac{\partial H_{\mathrm{pl}}}{\partial q_{\mathrm{c}}}\bigg|_{R_\ddagger,q_\ddagger} = \omega_{\mathrm{c}}^2\Big(q_\ddagger + \sqrt{\frac{2}{\hbar\omega_{\mathrm{c}}^3}}\chi \cdot \mu(R_\ddagger)\Big) = 0. \tag{20}$$

From Supplementary Eq. 20, we have $q_\ddagger = -\sqrt{\frac{2}{\hbar\omega_{\mathrm{c}}^3}}\chi\mu(R_\ddagger)$, plugging this back to Supplementary Eq. 19, we have $\frac{\partial H_{\mathrm{pl}}}{\partial R}\Big|_{R_\ddagger,q_\ddagger} = \frac{\partial E(R)}{\partial R}\Big|_{R_\ddagger} = 0$, *i.e.*, H_{pl} and $E(R)$ share the same saddle point (as well as same minimum as shown before). At $R_\ddagger$, the curvature of the reaction barrier is $M\omega_{\mathrm{b}}^2 = -\frac{\partial^2 E(R)}{\partial R^2}|_{R_\ddagger}$, and ω_{b} is the barrier frequency. We further denote the molecular dipole and

its derivative at $R_\ddagger$ as $\mu_\ddagger \equiv \mu(R_\ddagger)$ and $\mu'_\ddagger \equiv \frac{\partial \mu(R)}{\partial R}\Big|_{R_\ddagger}$, respectively. At $R = R_\ddagger$ and $q_\mathrm{c} = q_\ddagger$, the Hessian $\frac{\partial^2 \hat{H}_\mathrm{pl}}{\partial R^2}$ is evaluated as

$$\begin{aligned}\frac{\partial^2 \hat{H}_\mathrm{pl}}{\partial R^2}\bigg|_{R_\ddagger,q_\ddagger} &= \frac{\partial E^2(R)}{\partial R^2}\bigg|_{R_\ddagger} + \sqrt{\frac{2\omega_c}{\hbar}}\chi q_\mathrm{c}\,\frac{\partial \mu^2(R)}{\partial R^2}\bigg|_{R_\ddagger,q_\ddagger} + \frac{2\chi^2}{\hbar\omega_\mathrm{c}}\left[\mu(R)\,\frac{\partial \mu^2(R)}{\partial R^2}\bigg|_{R_0} + \left(\frac{\partial \mu(R)}{\partial R}\bigg|_{R_0}\right)^2\right] \\ &= -M\omega_\mathrm{b}^2 + \frac{2\chi^2}{\hbar\omega_c}{\mu'_\ddagger}^2. \end{aligned} \tag{21}$$

The other terms are also straight-forward to evaluate, resulting in

$$\mathcal{H}_\ddagger = \mathcal{H}(R_\ddagger, q_\ddagger) = \begin{pmatrix} -\omega_\mathrm{b}^2 + \frac{\mathcal{C}_\ddagger^2}{\omega_\mathrm{c}^2} & \mathcal{C}_\ddagger \\ \mathcal{C}_\ddagger & \omega_\mathrm{c}^2 \end{pmatrix}, \tag{22}$$

where $\mathcal{C}_\ddagger = \sqrt{\frac{2\omega_\mathrm{c}}{M\hbar}}\chi \cdot \mu'_\ddagger$. The normal mode frequencies $\tilde{\omega}$ are obtained by diagonalizing Supplementary Eq. 22, resulting in

$$\begin{aligned}\Omega_\pm^{\ddagger 2} &= \frac{1}{2}(-\omega_\mathrm{b}^2 + \frac{\mathcal{C}_\ddagger^2}{\omega_\mathrm{c}^2} + \omega_\mathrm{c}^2) \pm \frac{1}{2}\sqrt{(-\omega_\mathrm{b}^2 + \frac{\mathcal{C}_\ddagger^2}{\omega_\mathrm{c}^2} + \omega_\mathrm{c}^2)^2 + 4\omega_\mathrm{c}^2\omega_\mathrm{b}^2} \\ &= \frac{1}{2}(-\omega_\mathrm{b}^2 + \frac{\mathcal{C}_\ddagger^2}{\omega_\mathrm{c}^2} + \omega_\mathrm{c}^2) \pm \frac{1}{2}\sqrt{(-\omega_\mathrm{b}^2 + \frac{\mathcal{C}_\ddagger^2}{\omega_\mathrm{c}^2} - \omega_\mathrm{c}^2)^2 + 4\mathcal{C}_\ddagger^2}\end{aligned} \tag{23}$$

Supplementary Note 3. Model Molecular Hamiltonian

The potential energy surface (PES) and permanent dipole moment are taken from a Shin-Metiu model,[11] which is illustrated in Fig. 1 of the main text. The Shin-Metiu model is an one dimensional molecular system that describes a proton-coupled electron transfer reaction between a donor and an acceptor ion. The model consists of a transferring proton with a mass of m_p and charge z_p, an electron, and two fixed ions (a donor and an acceptor ion, with the charge of z_D and z_A, respectively). The molecular Hamiltonian is $\hat{H}_\mathrm{M} = \frac{\hat{P}^2}{2M} + \hat{H}_\mathrm{el} + \hat{H}_\mathrm{vib}$, where M is the mass of the nuclei (proton in this model), $\hat{H}_\mathrm{el} = \hat{T}_r + \hat{V}_\mathrm{eN} + \hat{V}_\mathrm{NN}$ is the electronic Hamiltonian, where $\hat{T}_r = \hat{p}_\mathrm{r}^2/2m_\mathrm{e}$ represents the kinetic energy operator of the electron with mass m_e, $\hat{V}_\mathrm{eN}$ describes the interaction between the electron and the three nuclei, which is written as a modified Coulomb

potential

$$\hat{V}_{\mathrm{eN}} = -z_{\mathrm{p}} e^2 \frac{\mathrm{erf}(\frac{|r-R|}{R_{\mathrm{c}}})}{|r-R|} - \sum_{\alpha\in\mathrm{D,A}} z_\alpha e^2 \frac{\mathrm{erf}(\frac{|r-R_\alpha|}{R_{\mathrm{c}}})}{|r-R_\alpha|}, \tag{24}$$

where r is the position of the electron and $e = 1$ a.u. is the fundamental charge, R is the position of the proton, while R_{D} and R_{A} are the positions of the donor and acceptor ion, respectively. R_{c} is a parameter that controls the strength of the modified Coulomb potentials. The nucleus-nucleus interaction potential V_{NN} that describes the Coulomb repulsion between the proton and the static ions takes the form of

$$V_{\mathrm{NN}} = \frac{z_{\mathrm{p}} z_{\mathrm{D}} e^2}{|R-R_{\mathrm{D}}|} + \frac{z_{\mathrm{p}} z_{\mathrm{A}} e^2}{|R-R_{\mathrm{A}}|} \tag{25}$$

The parameters in the molecular Hamiltonian $\hat{H}_{\mathrm{M}}$ used in this work is tabulated in Supplementary Table. 1. The resulting PES $E(R) = \langle \Psi_g(R)|(\hat{H}_{\mathrm{M}} - \hat{T} - \hat{H}_{\mathrm{vib}})|\Psi_g(R)\rangle$ and the permanent dipole moment $\mu(R) = \langle \Psi_g(R)|\hat{\mu}|\Psi_g(R)\rangle$ are shown in Fig. 1b and Fig. 1c, respectively.

Supplementary Table 1: Parameters used in the molecular Hamiltonian $\hat{H}_{\mathrm{M}}$.

Parameter	Value (unit)
z_{p} , z_{D}, z_{A}	1 (unitless)
R_{D}	-2.5 (Å)
R_{A}	2.5 (Å)
R_{c}	0.8 (Å)
m_{p}	1836 (a.u.)
m_{e}	1 (a.u.)

The vibrational Hamiltonian $\hat{H}_{\mathrm{vib}}$ is modeled by the Caldeira-Leggett[12] system-bath Hamiltonian

$$\hat{H}_{\mathrm{vib}} = \sum_k \frac{P_k^2}{2M_k} + \frac{M_k \omega_k^2}{2}(R_k + \frac{c_k}{M_k \omega_k^2} R)^2 \tag{26}$$

describes the interaction between the solvent mode R_{s} and a dissipative bath, where R_k represents the k_{th} bath mode with a conjugate momentum P_k and a mass $M_k = M_{\mathrm{s}}$. The coupling constant c_k and the frequency ω_k is characterized by an ohmic spectral density

$$J(\omega) = \frac{\pi}{2} \sum_k \frac{c_k^2}{M_k \omega_k} \delta(\omega - \omega_k) = \zeta \omega e^{-\omega/\omega_{\mathrm{p}}}, \tag{27}$$

with a characteristic phonon frequency ω_{p} and a friction constant ζ. The vibrational frequencies

and the coupling coefficients are sampled based on the following expressions[13]

$$\omega_k = -\omega_{\rm p}\ln(1 - k\frac{\omega_{\mathcal{N}}}{\omega_{\rm p}}); \quad c_k = \sqrt{\frac{2\zeta}{\pi}M_k\omega_{\mathcal{N}}}\,\omega_k, \tag{28}$$

where $\omega_{\mathcal{N}} = \frac{\omega_{\rm p}}{\mathcal{N}}(1 - e^{-\omega_{\rm m}/\omega_{\rm p}})$. The index k refers to the $k_{\rm th}$ bath mode, with $M_k = M = 1836$ a.u., and $\mathcal{N}$ represents the total number of total bath modes, and the maximum bath frequency is $\omega_{\rm m}$. In this work, we use $\mathcal{N} = 80$ bath modes and we choose the characteristic phonon bath frequency $\omega_{\rm p} = \omega_0 = 170.6$ meV, the maximum bath frequency $\omega_{\rm m} = 5\omega_{\rm p}$, and the friction constant, $\zeta = 49.6$ meV (400 cm^{-1}).

Supplementary Note 4. Derivation of the $\kappa_{\rm GH}$ from the Generalized Langevin Equation

Here, we provide an alternative derivation of the GH theory based upon the generalized Langevin equation[14–16] (GLE). We begin by expressing the total Hamiltonian in Eq. 1 of the main text as follows

$$H(R, q_{\rm c}, \{R_k\}) = \frac{P^2}{2M} + E(R) + \frac{p_{\rm c}^2}{2} + \frac{1}{2}\omega_{\rm c}^2\big(q_{\rm c} + \sqrt{\frac{2}{\hbar\omega_{\rm c}^3}}\chi\mu(R)\big)^2 + \sum_k \frac{P_k^2}{2M_k} + \frac{1}{2}M_k\omega_k^2\big(R_k + \frac{c_k}{M_k\omega_k^2}R\big)^2. \tag{29}$$

We further make the approximation of the permanent dipole by expanding it at the saddle point $R = R_{\ddagger}$ as

$$\mu(R) \approx \mu_{\ddagger} + \mu'_{\ddagger}\cdot(R - R_{\ddagger}) = \mu'_{\ddagger}\cdot R, \tag{30}$$

due to the fact that $\mu(R_{\ddagger}) = 0$ and $R_{\ddagger} = 0$ for the model system used in this work. In this work we obtain $\mu'_{\ddagger} = -1.887$ a.u. by fitting $\mu(R)$ in the vicinity of $R_{\ddagger}$ (see Supplementary Fig. 2). In addition, we expanding the potential energy of the molecule at $R = R_{\ddagger}$ as

$$E(R) \approx E(R_{\ddagger}) + \frac{\partial E(R)}{\partial R}\bigg|_{R_{\ddagger}}\cdot(R - R_{\ddagger}) + \frac{1}{2}\frac{\partial^2 E(R)}{\partial R^2}\bigg|_{R_{\ddagger}}\cdot(R - R_{\ddagger})^2 = E(R_{\ddagger}) - \frac{1}{2}M\omega_b^2R^2, \tag{31}$$

where we used the results that $\frac{\partial E(R)}{\partial R}\Big|_{R_{\ddagger}} = 0$ and $R_{\ddagger} = 0$.

To simply the derivation we use the mass-weighted coordinates and momentum, such that $X = \sqrt{M}R$ with $\Pi = \dot{X}$, $X_k = \sqrt{M_k}R_k = \sqrt{M}R_k$ with $\Pi_k = \dot{X}_k$ (where we use $M_k = M$) in this work. With the approximations in Supplementary Eq. 30 and Supplementary Eq. 31 as well as the mass-weighted coordinates and momenta, the Hamiltonian in Supplementary Eq. 29 is expressed as

$$H(X, q_{\rm c}, \{X_k\}) = \frac{\Pi^2}{2} - \frac{1}{2}\omega_{\rm b}^2 X^2 + \frac{p_{\rm c}^2}{2} + \frac{1}{2}\omega_{\rm c}^2\Big(q_{\rm c} + \sqrt{\frac{2}{M\hbar\omega_{\rm c}^3}}\chi\mu'_{\ddagger}X\Big)^2 + \sum_k \Big[\frac{\Pi_k^2}{2} + \frac{1}{2}\omega_k^2\Big(X_k + \frac{c_k}{M\omega_k^2}X\Big)^2\Big]$$
$$= \frac{\Pi^2}{2} - \frac{1}{2}\omega_{\rm b}^2 X^2 + \frac{p_{\rm c}^2}{2} + \frac{1}{2}\omega_{\rm c}^2\Big(q_{\rm c} + \frac{\mathcal{C}_{\ddagger}}{\omega_{\rm c}^2}X\Big)^2 + \sum_k \Big[\frac{\Pi_k^2}{2} + \frac{1}{2}\omega_k^2\Big(X_k + \frac{\mathcal{C}_k}{\omega_k^2}X\Big)^2\Big], \tag{32}$$

where in the second line we introduced

$$\mathcal{C}_{\ddagger} = \sqrt{\frac{2\omega_{\rm c}}{M\hbar}}\chi\mu'_{\ddagger}; \quad \mathcal{C}_k = \frac{c_k}{\sqrt{MM_k}}, \tag{33}$$

and c_k is defined in Supplementary Eq. 27-28. The Hamilton's equations of motion of $H(X, q_{\rm c}, \{X_k\})$ are

$$\dot{X} = \frac{\partial H}{\partial \Pi} = \Pi, \quad \dot{X}_k = \frac{\partial H}{\partial \Pi_k} = \Pi_k, \quad \dot{q}_c = \frac{\partial H}{\partial p_{\rm c}} = p_{\rm c}, \tag{34}$$
$$\ddot{X} = \dot{\Pi} = -\frac{\partial H}{\partial X} = \omega_{\rm b}^2 X - \mathcal{C}_{\ddagger}\Big(q_{\rm c} + \frac{\mathcal{C}_{\ddagger}}{\omega_{\rm c}^2}X\Big) - \sum_k \mathcal{C}_k\Big(X_k + \frac{\mathcal{C}_k}{\omega_k^2}X\Big) \tag{35}$$
$$\ddot{X}_k = \dot{\Pi}_k = -\frac{\partial H}{\partial X_k} = -\omega_k^2\Big(X_k + \frac{\mathcal{C}_k}{\omega_k^2}X\Big) \tag{36}$$
$$\ddot{q}_{\rm c} = \dot{p}_{\rm c} = -\frac{\partial H}{\partial q_{\rm c}} = -\omega_{\rm c}^2\Big(q_{\rm c} + \frac{\mathcal{C}_{\ddagger}}{\omega_{\rm c}^2}X\Big). \tag{37}$$

To solve the above coupled equations, we perform the Laplace transform of function $f(t)$ as $\tilde{f}(\gamma) = \mathcal{L}[f(t)] = \int_0^\infty dt e^{-\gamma t} f(t)$. The Laplace transform of $\ddot{X}$, $\ddot{X}_k$ and $\ddot{q}_{\rm c}$ are given as

$$\tilde{\ddot{X}}(\gamma) = -\Pi(0) - \gamma X(0) + \gamma^2 \tilde{X}(\gamma) \tag{38}$$

$$\tilde{\ddot{X}}_k(\gamma) = -\Pi_k(0) - \gamma X_k(0) + \gamma^2 \tilde{X}_k(\gamma) \tag{39}$$

$$\tilde{\ddot{q}}_{\rm c}(\gamma) = -p_{\rm c}(0) - \gamma q_{\rm c}(0) + \gamma^2 \tilde{q}_{\rm c}(\gamma) \tag{40}$$

Using the above expressions in Supplementary Eq. 36-37, and plugging them back into the Laplace transformed Eq.36-37, we have

$$\tilde{X}_k(\gamma) = \frac{\Pi_k(0) + \gamma X_k(0)}{\gamma^2 + \omega_k^2} - \frac{\mathcal{C}_k \tilde{X}(\gamma)}{\gamma^2 + \omega_k^2}, \tag{41}$$

$$\tilde{q}_\mathrm{c}(\gamma) = \frac{p_\mathrm{c}(0) + \gamma q_\mathrm{c}(0)}{\gamma^2 + \omega_\mathrm{c}^2} - \frac{\mathcal{C}_\ddagger \tilde{X}(\gamma)}{\gamma^2 + \omega_\mathrm{c}^2}. \tag{42}$$

The Laplace transformed Supplementary Eq. 35 can be expressed as

$$\begin{aligned}
&-\Pi(0) - \gamma X(0) + \gamma^2 \tilde{X}(\gamma) = \Big(\omega_\mathrm{b}^2 - \frac{\mathcal{C}_\ddagger^2}{\omega_\mathrm{c}^2} - \sum_k \frac{\mathcal{C}_k^2}{\omega_\mathrm{c}^2}\Big)\tilde{X}(\gamma) - \mathcal{C}_\ddagger \tilde{q}_\mathrm{c}(\gamma) - \sum_k \mathcal{C}_k \tilde{X}_k(\gamma) \qquad (43)\\
&= \Big(\omega_\mathrm{b}^2 - \frac{\mathcal{C}_\ddagger^2}{\omega_\mathrm{c}^2} - \sum_k \frac{\mathcal{C}_k^2}{\omega_\mathrm{c}^2}\Big)\tilde{X}(\gamma) + \frac{\mathcal{C}_\ddagger^2 \tilde{X}(\gamma)}{\gamma^2 + \omega_\mathrm{c}^2} + \sum_k \frac{\mathcal{C}_k^2 \tilde{X}(\gamma)}{\gamma^2 + \omega_k^2} - \mathcal{C}_\ddagger \frac{p_\mathrm{c}(0) + \gamma q_\mathrm{c}(0)}{\gamma^2 + \omega_\mathrm{c}^2} - \sum_k \mathcal{C}_k \frac{\Pi_k(0) + \gamma X_k(0)}{\gamma^2 + \omega_k^2}\\
&= \omega_\mathrm{b}^2 \tilde{X}(\gamma) - \frac{\mathcal{C}_\ddagger^2}{\omega_\mathrm{c}^2}\Big(\frac{\gamma}{\gamma^2 + \omega_\mathrm{c}^2}\Big)\gamma\tilde{X}(\gamma) - \sum_k \frac{\mathcal{C}_k^2}{\omega_k^2}\Big(\frac{\gamma}{\gamma^2 + \omega_k^2}\Big)\gamma\tilde{X}(\gamma) - \mathcal{C}_\ddagger \frac{p_\mathrm{c}(0) + \gamma q_\mathrm{c}(0)}{\gamma^2 + \omega_\mathrm{c}^2} - \sum_k \mathcal{C}_k \frac{\Pi_k(0) + \gamma X_k(0)}{\gamma^2 + \omega_k^2},
\end{aligned}$$

where we have used Supplementary Eq. 41-42, Supplementary Eq. 38, as well as the fact that $-\frac{\mathcal{C}_\ddagger^2}{\omega_\mathrm{c}^2}\tilde{X}(\gamma) + \frac{\mathcal{C}_\ddagger^2 \tilde{X}(\gamma)}{\gamma^2+\omega_\mathrm{c}^2} = -\frac{\mathcal{C}_\ddagger^2}{\omega_\mathrm{c}^2}\Big(\frac{\gamma}{\gamma^2+\omega_\mathrm{c}^2}\Big)\gamma\tilde{X}(\gamma)$ and $-\frac{\mathcal{C}_k^2}{\omega_k^2}\tilde{X}(\gamma) + \frac{\mathcal{C}_k^2 \tilde{X}(\gamma)}{\gamma^2+\omega_k^2} = -\frac{\mathcal{C}_k^2}{\omega_k^2}\Big(\frac{\gamma}{\gamma^2+\omega_k^2}\Big)\gamma\tilde{X}(\gamma)$.

We further define the Laplace transformed frictions $\tilde{\xi}_\mathrm{c}(\gamma)$ (cavity photon friction) and $\tilde{\xi}_\mathrm{p}(\gamma)$ (phonon bath friction) as well as the random noise $\tilde{\mathcal{F}}_\mathrm{c}(\gamma)$ (cavity photon noise) and $\tilde{\mathcal{F}}_\mathrm{p}(\gamma)$ (phonon bath noise) as follows

$$\tilde{\xi}_\mathrm{c}(\gamma) = \frac{\mathcal{C}_\ddagger^2}{\omega_\mathrm{c}^2}\Big(\frac{\gamma}{\gamma^2 + \omega_\mathrm{c}^2}\Big) \tag{44}$$

$$\tilde{\xi}_\mathrm{p}(\gamma) = \sum_k \frac{\mathcal{C}_k^2}{\omega_k^2}\Big(\frac{\gamma}{\gamma^2 + \omega_k^2}\Big) \tag{45}$$

$$\tilde{\mathcal{F}}_\mathrm{c}(\gamma) = -\mathcal{C}_\ddagger \frac{p_\mathrm{c}(0) + \gamma q_\mathrm{c}(0)}{\gamma^2 + \omega_\mathrm{c}^2} \tag{46}$$

$$\tilde{\mathcal{F}}_\mathrm{p}(\gamma) = -\sum_k \mathcal{C}_k \frac{\Pi_k(0) + \gamma X_k(0)}{\gamma^2 + \omega_k^2}. \tag{47}$$

Using the above definitions in Supplementary Eq. 43, we have

$$-\Pi(0) - \gamma X(0) + \gamma^2 \tilde{X}(\gamma) = \omega_\mathrm{b}^2 \tilde{X}(\gamma) - \tilde{\xi}_\mathrm{c}(\gamma)\gamma\tilde{X}(\gamma) - \tilde{\xi}_\mathrm{p}(\gamma)\gamma\tilde{X}(\gamma) + \tilde{\mathcal{F}}_\mathrm{c}(\gamma) + \tilde{\mathcal{F}}_\mathrm{p}(\gamma) \tag{48}$$

We take the inverse Laplace transform of Supplementary Eq. 48 as follows

$$\mathcal{L}^{-1}[\tilde{\ddot{X}}(\gamma)] = \omega_{\mathrm{b}}^2\mathcal{L}^{-1}[\tilde{X}(\gamma)] - \mathcal{L}^{-1}[\tilde{\xi}_{\mathrm{c}}(\gamma)\gamma\tilde{X}(\gamma)] - \mathcal{L}^{-1}[\tilde{\xi}_{\mathrm{p}}(\gamma)\gamma\tilde{X}(\gamma)] + \mathcal{L}^{-1}[\tilde{\mathcal{F}}_{\mathrm{c}}(\gamma)] + \mathcal{L}^{-1}[\tilde{\mathcal{F}}_{\mathrm{p}}(\gamma)],$$

where we denote the inverse Laplace transform as $\mathcal{L}^{-1}[\tilde{f}(\gamma)] = f(t)$. Each term in the above equation are inverse Laplace transformed as follows

$$\mathcal{L}^{-1}[-\Pi(0) - \gamma X(0) + \gamma^2\tilde{X}(\gamma)] = \mathcal{L}^{-1}[\tilde{\ddot{X}}(\gamma)] = \ddot{X}, \tag{49}$$

$$\mathcal{L}^{-1}[\tilde{X}(\gamma)] = X \tag{50}$$

$$\xi_{\mathrm{c}}(t) = \frac{\mathcal{C}_{\ddagger}^2}{\omega_{\mathrm{c}}^2}\mathcal{L}^{-1}\left[\frac{\gamma}{\gamma^2+\omega_{\mathrm{c}}^2}\right] = \frac{\mathcal{C}_{\ddagger}^2}{\omega_{\mathrm{c}}^2}\cos(\omega_{\mathrm{c}}t) \tag{51}$$

$$\xi_{\mathrm{p}}(t) = \sum_k \frac{\mathcal{C}_k^2}{\omega_k^2}\mathcal{L}^{-1}\left[\frac{\gamma}{\gamma^2+\omega_k^2}\right] = \sum_k \frac{\mathcal{C}_k^2}{\omega_k^2}\cos(\omega_k t) \tag{52}$$

$$\mathcal{F}_{\mathrm{c}}(t) = \mathcal{L}^{-1}[\tilde{\mathcal{F}}_{\mathrm{c}}(\gamma)] = -\mathcal{C}_{\ddagger}\Big(\frac{p_{\mathrm{c}}(0)}{\omega_c}\sin(\omega_{\mathrm{c}}t) + q_{\mathrm{c}}(0)\cos(\omega_{\mathrm{c}}t)\Big) \tag{53}$$

$$\mathcal{F}_{\mathrm{p}}(t) = \mathcal{L}^{-1}[\tilde{\mathcal{F}}_{\mathrm{p}}(\gamma)] = -\sum_k \mathcal{C}_k\Big(\frac{\Pi_k(0)}{\omega_k}\sin(\omega_k t) + X_k(0)\cos(\omega_k t)\Big) \tag{54}$$

where in the second and third line we have used $\mathcal{L}^{-1}[\gamma\tilde{X}(\gamma)](\tau) = \dot{X}(t)$. Further, using the convolution theorem, we have

$$\mathcal{L}^{-1}[\tilde{\xi}_{\mathrm{c}}(\gamma)\gamma\tilde{X}(\gamma)] = \int_0^t \mathcal{L}^{-1}[\tilde{\xi}_{\mathrm{c}}(\gamma)](\tau)\cdot\mathcal{L}^{-1}[\gamma\tilde{X}(\gamma)](t-\tau)d\tau = \int_0^t \xi_{\mathrm{c}}(\tau)\cdot\dot{X}(t-\tau)d\tau \tag{55}$$

$$\mathcal{L}^{-1}[\tilde{\xi}_{\mathrm{p}}(\gamma)\gamma\tilde{X}(\gamma)] = \int_0^t \mathcal{L}^{-1}[\tilde{\xi}_{\mathrm{p}}(\gamma)](\tau)\cdot\mathcal{L}^{-1}[\gamma\tilde{X}(\gamma)](t-\tau)d\tau = \int_0^t \xi_{\mathrm{p}}(\tau)\cdot\dot{X}(t-\tau)d\tau. \tag{56}$$

Using Supplementary Eq. 49-56, we arrive at the following generalized Langevin equation (GLE) for the mass weighted coordinate X as follows

$$\ddot{X} = \omega_{\mathrm{b}}^2 X - \int_0^t \big(\xi_{\mathrm{c}}(\tau) + \xi_{\mathrm{p}}(\tau)\big)\cdot\dot{X}(t-\tau)d\tau + \mathcal{F}_{\mathrm{c}}(t) + \mathcal{F}_{\mathrm{p}}(t), \tag{57}$$

where $\mathcal{F}_{\mathrm{c}}(t)$ and $\mathcal{F}_{\mathrm{p}}(t)$ are the total random noise associated with the cavity mode and phonon bath, respectively, each satisfies fluctuation-dissipation theorem as $\langle\mathcal{F}_{\mathrm{c}}(0)\mathcal{F}_{\mathrm{c}}(t)\rangle = Mk_{\mathrm{B}}T\xi_{\mathrm{c}}(t)$ and

$\langle \mathcal{F}_{\rm p}(0)\mathcal{F}_{\rm p}(t)\rangle = Mk_{\rm B}T\xi_{\rm p}(t)$. In the original coordinate R, the GLE is expressed as

$$\boxed{\ddot{R} = \omega_{\ddagger}^2 R - \int_0^t \big(\xi_{\rm c}(\tau) + \xi_{\rm p}(\tau)\big)\cdot \dot{R}(t-\tau)d\tau + \frac{1}{\sqrt{M}}\big(\mathcal{F}_{\rm c}(t) + \mathcal{F}_{\rm p}(t)\big)}. \tag{58}$$

For the simplified model system of $\hat{H} - \hat{H}_{\rm vib}$, the GLE is expressed as

$$\ddot{R} = \omega_{\ddagger}^2 R - \int_0^t \xi_{\rm c}(\tau)\cdot \dot{R}(t-\tau)d\tau + \frac{1}{\sqrt{M}}\mathcal{F}_{\rm c}(t). \tag{59}$$

Using the above generalized Langevin equation in Supplementary Eq. 58, we can obtain a rate constant expression (transmission coefficient), which is commonly referred to as the Grote-Hynes (GH) rate theory. The trajectory $R(t)$ is reactive if $R(t) > 0$ for $t \to \infty$. Assuming that $R(t) = R(0)e^{\lambda t}$, where λ indicates the frequency of the trajectories crossing the top of the barrier, and plugging it into Supplementary Eq. 58, we have

$$\lambda^2 R(0)e^{\lambda t} = \omega_{\ddagger}^2 R(0)e^{\lambda t} - \lambda R(0)e^{\lambda t}\int_0^\infty \big(\xi_{\rm c}(\tau) + \xi_{\rm p}(\tau)\big)\cdot e^{-\lambda\tau}d\tau + \frac{1}{\sqrt{M}}\big(\mathcal{F}_{\rm c}(t) + \mathcal{F}_{\rm p}(t)\big). \tag{60}$$

Using the fact that $\langle \mathcal{F}_{\rm p}(t)\rangle = \langle \mathcal{F}_{\rm c}(t)\rangle = 0$, taking the ensemble average of the above equation over the initial distribution $R(0)$, and recognize that $\int_0^\infty \big(\xi_{\rm c}(\tau) + \xi_{\rm p}(\tau)\big)\cdot e^{-\lambda\tau}d\tau = \tilde{\xi}_{\rm c}(\lambda) + \tilde{\xi}_{\rm p}(\lambda)$, we have the following Grote-Hynes equation for the frequency λ as follows

$$\boxed{\lambda^2 = \omega_{\rm b}^2 - \lambda\cdot(\tilde{\xi}_{\rm c}(\lambda) + \tilde{\xi}_{\rm p}(\lambda))}, \tag{61}$$

where $\tilde{\xi}_{\rm c}(\gamma)$ and $\tilde{\xi}_{\rm p}(\gamma)$ are expressed in Supplementary Eq. 44 and Supplementary Eq. 45, respectively. The transmission coefficient in the GH rate theory (by using the positive solution of λ in Supplementary Eq. 61) is given as

$$\kappa_{\rm GH} = \frac{\lambda}{\omega_{\rm b}}. \tag{62}$$

The same result can also be derived using the normal-mode analysis of the Hamiltonian as shown in Ref. 17

For the model system considered in this study, Supplementary Eq. 61 needs to be solve numer-

ically. Using the spectral density $J(\omega) = \frac{\pi}{2}\sum_k \frac{c_k^2}{M_k\omega_k}\delta(\omega-\omega_k)$ One can explicitly consider $\tilde{\xi}_{\mathrm{p}}(\gamma)$ as follows

$$\tilde{\xi}_{\mathrm{p}}(\gamma) = \sum_k \frac{\mathcal{C}_k^2}{\omega_k^2}\Big(\frac{\gamma}{\gamma^2+\omega_k^2}\Big) = \frac{1}{M}\sum_k \frac{c_k^2}{M_k\omega_k^2}\Big(\frac{\gamma}{\gamma^2+\omega_k^2}\Big) = \frac{2}{M\pi}\int_0^\infty \frac{J(\omega)}{\omega}\frac{\gamma}{\gamma^2+\omega^2}d\omega. \tag{63}$$

Using Supplementary Eq. 63 and numerically solving Supplementary Eq. 61 gives the GH transmission coefficients of the model molecule-cavity hybrid system.

Alternatively, one can take the Markovian limit of the phonon bath $\hat{H}_{\mathrm{vib}}$. Note that

$$\xi_{\mathrm{p}}(t) = \frac{1}{M}\sum_k \frac{c_k^2}{M_k\omega_k^2}\cos(\omega_k t) = \frac{2}{\pi M}\int_0^\infty d\omega\frac{J(\omega)}{\omega}\cos(\omega t), \tag{64}$$

for the model system used in this work, $J(\omega) = \zeta\omega e^{-\omega/\omega_{\mathrm{p}}}$ (Supplementary Eq. 27), we take the Markovian limit $\omega_{\mathrm{p}} \to \infty$ (ω_{p} larger than all physical frequency of the system), which leads to

$$\xi_{\mathrm{p}}(t) = \lim_{\omega_{\mathrm{p}}\to\infty}\frac{2}{\pi M}\int_0^\infty d\omega\zeta e^{-\omega/\omega_{\mathrm{p}}}\cos(\omega t) = \lim_{\omega_{\mathrm{p}}\to\infty}\frac{2\zeta}{M}\frac{1}{\pi}\frac{\omega_{\mathrm{p}}}{1+(\omega_{\mathrm{p}}t)^2} = \frac{2\zeta}{M}\delta(t). \tag{65}$$

The Laplace transformed kernel under the Markovian limit is then

$$\tilde{\xi}_{\mathrm{p}}(\gamma) = \int_0^\infty dte^{-\gamma t}\xi_{\mathrm{p}}(t) = \frac{1}{2}\int_{-\infty}^\infty dte^{-\gamma t}\frac{2\zeta}{M}\delta(t) = \frac{\zeta}{M}, \tag{66}$$

using the above Markovian limit, Supplementary Eq. 61 is simplified into following equation of λ

$$\lambda^4 + \frac{\zeta}{M}\lambda^3 + \Big(\omega_{\mathrm{c}}^2 - \omega_{\mathrm{b}}^2 + \frac{\mathcal{C}_\ddagger^2}{\omega_{\mathrm{c}}^2}\Big)\lambda^2 + \frac{\zeta}{M}\omega_{\mathrm{c}}^2\lambda - \omega_{\mathrm{c}}^2\omega_{\mathrm{b}}^2 = 0. \tag{67}$$

which facilitate the procedure of solving λ numerically.

Finally, for the simplified model system with $\hat{H} - \hat{H}_{\mathrm{vib}}$, the GLE is expressed in Supplementary Eq. 59, and the GH equation of λ is

$$\lambda^2 = \omega_{\mathrm{b}}^2 - \lambda\frac{\mathcal{C}_\ddagger^2}{\omega_{\mathrm{c}}^2}\Big(\frac{\lambda}{\lambda^2+\omega_{\mathrm{c}}^2}\Big), \tag{68}$$

which is the same as

$$\lambda^4 + \Big(\omega_c^2 - \omega_b^2 + \frac{\mathcal{C}_\ddagger^2}{\omega_c^2}\Big)\lambda^2 - \omega_c^2\omega_b^2 = 0. \tag{69}$$

The analytical solution of the above equation is

$$\lambda^2 = -\frac{1}{2}\Big(\omega_c^2 - \omega_b^2 + \frac{\mathcal{C}_\ddagger^2}{\omega_c^2}\Big) \pm \frac{1}{2}\sqrt{\Big(\omega_c^2 - \omega_b^2 + \frac{\mathcal{C}_\ddagger^2}{\omega_c^2}\Big)^2 + 4\omega_b^2\omega_c^2} \tag{70}$$

Taking the positive solution, the analytical κ_{GH} has the following expression

$$\kappa_{\mathrm{GH}} = \frac{1}{\omega_b}\left[\frac{1}{2}\left(-\Delta\omega_\ddagger^2 + \sqrt{(\Delta\omega_\ddagger^2)^2 + 4\omega_b^2\omega_c^2}\right)\right]^{\frac{1}{2}}, \tag{71}$$

where $\Delta\omega_\ddagger^2 \equiv \omega_c^2 - \omega_b^2 + \frac{\mathcal{C}_\ddagger^2}{\omega_c^2}$, with $\mathcal{C}_\ddagger = \sqrt{\frac{2\omega_c}{M\hbar}}\chi \cdot \mu'_\ddagger$ and $M\omega_b^2 = -\frac{\partial^2 E(R)}{\partial R^2}|_{R_\ddagger}$, and $\mu'_\ddagger = \frac{\partial \mu}{\partial R}|_{R_\ddagger}$ is the slope of the dipole moment on the dividing surface $R_\ddagger$. This is the result presented in Eq. 5 of the main text.

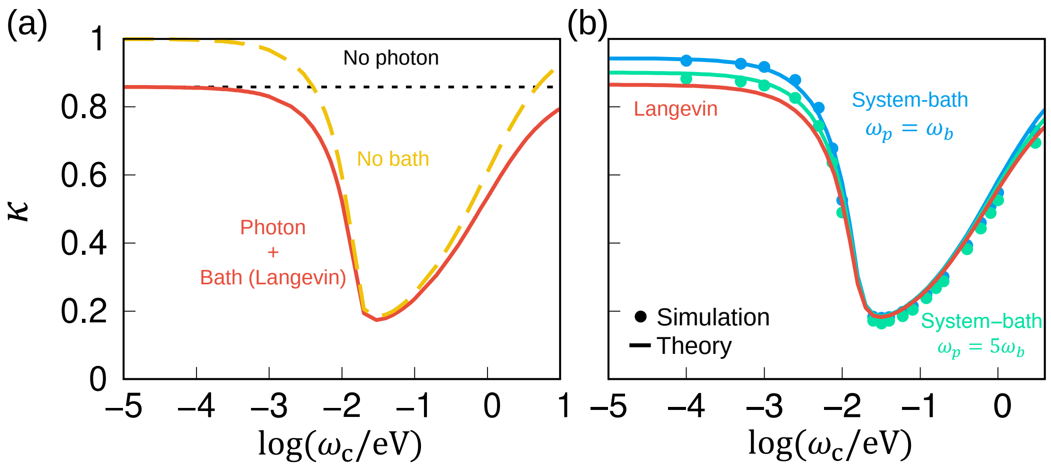

Supplementary Figure 1: Transmission coefficient κ_{GH} as a function of the photon frequency ω_c and with the light-matter coupling constant $\eta = 0.188$, computed under different treatment of the phonon bath $\hat{H}_{\mathrm{vib}}$. (a) The black dashed line corresponds to the results of the model system $\hat{H}_M$ *i.e.*, without coupling to the cavity. The yellow dashed curve represents the results of the model system $\hat{H} - \hat{H}_{\mathrm{vib}}$, without considering the phonon bath, indicated by the analytical result in Supplementary Eq. 71. The red solid curve represents the results of κ_{GH} with a Markovian limit of the phonon bath, by solving Supplementary Eq. 67. (b) κ_{GH} for the full model system obtained by numerically solving Supplementary Eq. 61. The characteristic frequency of the phonon bath ω_p is changed from $\omega_p = \omega_b$ (blue) to $\omega_p = 5\omega_b$ (green), compared to the Markovian limit (red solid curve, by solving Supplementary Eq. 67). The Markovian limit of the phonon bath is achieved when $\omega_p = 25\omega_b$. Filled circles are results obtained from direct numerical simulation of computing Eq. 4 of the main text, with the details in Supplementary Note 5.

Supplementary Fig. 1 presents the numerical values of $\kappa_{\rm GH}$ obtained by a various theoretical descriptions of the phonon bath $\hat{H}_{\rm vib}$. Here, we use $\eta = 0.188$, and $\mu'_{\ddagger}$ fitted in the region around the dividing surface (as shown in Supplementary Fig. 2. Supplementary Fig. 1 presents the influence of the cavity mode $q_{\rm c}$ and the phonon bath $\hat{H}_{\rm vib}$. The black dashed line corresponds to the results of the model system $\hat{H}_{\rm M}$ *i.e.*, without coupling to the cavity. The yellow dashed curve represents the results of the model system $\hat{H} - \hat{H}_{\rm vib}$, without considering the phonon bath, indicated by the analytical result in Supplementary Eq. 71. The red solid curve represents the results of $\kappa_{\rm GH}$ with a Langevin limit of the phonon bath, by solving Supplementary Eq. 67. One can clearly see that with the Langevin phonon bath (red curve) or without the phonon bath (yellow curve), the resonant behavior of $\kappa_{\rm GH}$ as a function of $\omega_{\rm c}$ remains the same. We have also tested with other η values used in the main text and the resonant behavior of κ remains invariant with the presence of the phonon bath. Note that all results presented in the main text were computed with thermal bath to obtain a stable plateau for the flux-side correlation function.

Supplementary Fig. 1b presents $\kappa_{\rm GH}$ for the full model system $\hat{H}$ obtained by numerically solving Supplementary Eq. 61. The characteristic frequency of the phonon bath $\omega_{\rm p}$ is changed from $\omega_{\rm p} = \omega_{\rm b}$ (blue) to $\omega_{\rm p} = 5\omega_{\rm b}$ (green), compared to the Langevin limit (red solid curve, by solving Supplementary Eq. 67). Filled circles are results obtained from direct numerical simulation of computing the flux-side correlation function (Eq. 4 of the main text), with details in Supplementary Note 5.d. Clearly, the non-Markovian behavior of the phonon bath $\hat{H}_{\rm vib}$ has a very limited influence on κ over the entire range of the cavity frequency $\omega_{\rm c}$.

Supplementary Note 5. Computational Details

a. Electronic Ground State and Dipole Moment. We use the Fourier Grid Hamiltonian (FGH) approach[18,19] to solve the eigenvalue problem of all matter and polariton Hamiltonians. In particular, we use a total of $N = 2000$ grid basis $\{|r_i\rangle\}$ to describe the electronic degrees of freedom r in the range $[R_{\rm D} - 10, R_{\rm A} + 10]$ (with $\Delta r =$ 0.01 a.u.), which allows us to solve the adiabatic states of the matter. The matrix elements of the electronic Hamiltonian $\hat{H}_{\rm el} = \hat{H}_{\rm M} - \hat{T} - \hat{H}_{\rm vib}$ in

this grid basis $\{|r_i\rangle\}$ are given by

$$\langle r_i|\hat{H}_{\text{el}}|r_j\rangle = \langle r_i|\hat{T}_r + \hat{V}_{\text{eN}}(\hat{r}, R) + \hat{V}_{\text{NN}}(R)|r_j\rangle = \langle r_i|\hat{T}_r|r_j\rangle + [\hat{V}_{\text{eN}}(r_j, R) + \hat{V}_{\text{NN}}(R)]\delta_{ij}, \tag{72}$$

where $\hat{V}_{\text{eN}}(\hat{r}, R)$ and $\hat{V}_{\text{NN}}(R)$ are defined in Eq. 24 and Eq. 25, respectively. Note that when computing the adiabatic electronic energies, the nuclear position R is viewed as a parameter.

Further, $\langle r_i|\hat{T}_r|r_j\rangle$ is given analytically[18,19] as follows

$$\langle r_i|\hat{T}_r|r_j\rangle = \frac{\hbar^2}{2m}\cdot\frac{\pi^2}{3(\Delta r)^2}\left(1+\frac{2}{N^2}\right)\delta_{ij} + \frac{\hbar^2}{2m}\cdot\frac{2(-1)^{j-i}\pi^2}{\left(\Delta r N \sin\left(\frac{\pi(j-i)}{N}\right)\right)^2}(1-\delta_{ij}). \tag{73}$$

Directly diagonalizing the matrix of $\langle r_i|\hat{H}_{\text{el}}|r_j\rangle$ at a given nuclear position R in this grid basis gives the adiabatic electronic states $\hat{H}_{\text{el}}|\Psi_g(R)\rangle = E(R)|\Psi_g(R)\rangle$. In this work, we only focus on the electronic ground state

$$|\Psi_g(R)\rangle = \sum_i c_i(R)|r_i\rangle, \tag{74}$$

where $c_i(R) = \langle r_i|\Psi_g(R)\rangle$ is the expansion coefficient obtained by diagonalizing the matrix of $\langle r_i|\hat{H}_{\text{el}}|r_j\rangle$. The ground state permanent dipole moment $\hat{\mu} = R + \hat{r}$ is computed as

$$\mu(R) = \langle\Psi_g(R)|\hat{\mu}|\Psi_g(R)\rangle = \langle\Psi_g|(R+\hat{r})|\Psi_g\rangle = R + \sum_i c_i^*(R)c_i(R)r_i. \tag{75}$$

The numerical results of $E(R)$ and $\mu(R)$ are presented in Fig. 1b and Fig. 1c of the main text.

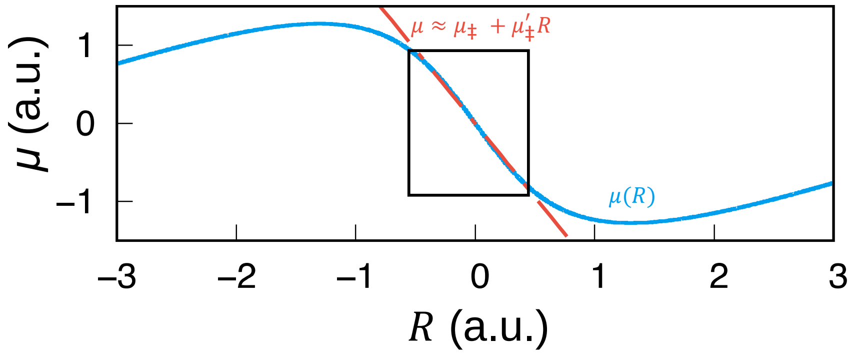

Supplementary Figure 2: Fitting permanent dipole moment at $R_\ddagger$ with $\mu = \mu_\ddagger + \mu'_\ddagger R$ for R in the region of [-0.5, 0.5]. The fitted $\mu'_\ddagger$ = -1.887 a.u. and $\mu_\ddagger$ = 0 a.u., which are used in the main text for computing the rate constant in the GH theory.

Supplementary Fig. 2 presents the permanent dipole moment. The red line indicates the linear fit of the dipole centered at $R = R_{\ddagger} = 0$. The fitted $\mu'_{\ddagger}$ = -1.887 a.u. and $\mu_{\ddagger} = 0$ a.u., which are used for computing the rate constant in the GH theory.

Supplementary Fig. 3 presents the adiabatic electronic potential $E_\alpha(R)$ (bold curves) by solving $\hat{H}_{\rm el}|\Psi_\alpha\rangle = E_\alpha(R)|\Psi_\alpha\rangle$, as well as photon dressed potentials $E_\alpha(R) + (n + \frac{1}{2})\hbar\omega_{\rm c}$ (thin curves). One can clearly see that the the ground adiabatic surface (black thick curve) is well separated from the first excited adiabatic electronic surface (blue thick curve) by at least 3.5 eV, and the photon dressed state $|\Psi_g, 30\rangle$ is at the same energy of $|\Psi_{\rm S1}, 0\rangle$. However, they are not directly coupled to each other from the light matter interactions. Thus the presence of electronic excited states has a negligible influence on the polariton absorption spectrum and the reaction dynamics.

We emphasize that it is crucial to consider the dipole self-energy (DSE) term $\frac{\omega_{\rm c}}{\hbar}(\hat{\boldsymbol{\mu}}\mathbf{A}_0)^2$ in Supplementary Eq. 10. Ignoring this term[20] will cause an artificial change of the barrier height on the Cavity Born-Oppenheimer Surface defined as $E_{\rm b} = E(R_{\ddagger}) - E(R_0)$, which is demonstrated in Supplementary Fig. 3(b), resulting in a large error of the rate constant.

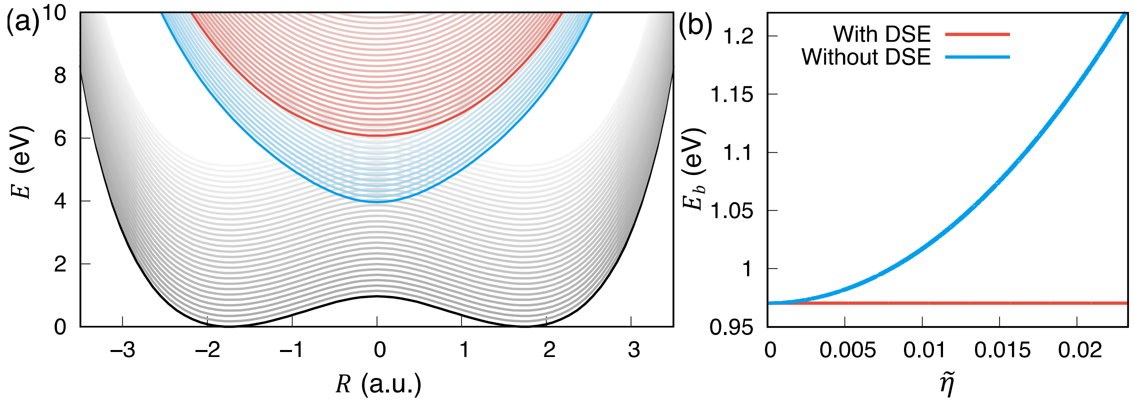

Supplementary Figure 3: (a) The adiabatic potential energy surface corresponding to the first three electronic states $|\Psi_g\rangle$ (black bold line), $|\Psi_{\rm S1}\rangle$ (blue bold line) and $|\Psi_{\rm S2}\rangle$ (red bold line). On top of these electronic adiabatic surfaces, 30 photon-dressed states are depicted on top of each electronic adiabatic surface. (b) $E_{\rm b} = E(R_{\ddagger}) - E(R_0)$ along the minimum energy path as a function of $\tilde{\eta}$ when $\omega_c = 0.17$ eV. As the coupling strength increases, ignoring DSE results in a rise of the potential energy barrier height.

b. Polariton Eigenspectrum and Absorption Spectrum. The vibrational polaritonic Hamiltonian is defined as follows

$$\begin{aligned}\hat{H}_{\mathrm{vpl}} &= \frac{\hat{P}^2}{2M} + E(\hat{R}) + \frac{1}{2}\hat{p}_{\mathrm{c}}^2 + \frac{1}{2}\omega_{\mathrm{c}}^2(\hat{q}_{\mathrm{c}} + \frac{A_0\mu(\hat{R})}{\sqrt{\hbar\omega_{\mathrm{c}}}})^2 \\ &= \frac{\hat{P}^2}{2M} + E(\hat{R}) + \hbar\omega_{\mathrm{c}}(\hat{a}^\dagger\hat{a} + \frac{1}{2}) + A_0\omega_{\mathrm{c}}\hat{\mu}(\hat{a} + \hat{a}^\dagger) + \frac{\omega_{\mathrm{c}}}{\hbar}(A_0\mu(\hat{R})^2),\end{aligned} \tag{76}$$

where $E(\hat{R}) = \langle\Psi_g(R)|(\hat{H}_{\mathrm{M}} - \hat{T} - \hat{H}_{\mathrm{vib}})|\Psi_g(R)\rangle$ is the ground state adiabatic surface, $\mu(\hat{R}) = \langle\Psi_g(R)|\hat{\mu}|\Psi_g(R)\rangle$ is the ground state permanent dipole obtained from Eq. 75. In this Hamiltonian, we treat the nuclear DOF quantum mechanically. We numerically obtain the vibrational polariton eigenvalue $\mathcal{E}_\nu$ and eigenvector $\{|\Phi_\nu\rangle\}$ by solving the following eigenequation

$$\hat{H}_{\mathrm{vpl}}|\Phi_\nu\rangle = \mathcal{E}_\nu|\Phi_\nu\rangle \tag{77}$$

using the R-grid-Fock basis $\{|R_j\rangle \otimes |n\rangle \equiv |R_j, n\}$, where $\{|R_j\rangle\}$ are the nuclear grid points, $|\{n\rangle\}$ are vacuum Fock states, *i.e.*, the eigenstates of the photonic Hamiltonian $\hat{H}_{\mathrm{ph}} = (\hat{a}^\dagger\hat{a} + \frac{1}{2})\hbar\omega_{\mathrm{c}}$. The matrix elements of the vibrational polaritonic Hamiltonian $\hat{H}_{\mathrm{vpl}}$ is given as

$$\begin{aligned}\langle m|\langle R_i|\hat{H}_{\mathrm{vpl}}|R_j\rangle|n\rangle &= \langle R_i|\hat{T}_R|R_j\rangle\delta_{nm} + E(R_j)\delta_{ij}\delta_{nm} + \big(n + \frac{1}{2}\big)\hbar\omega_{\mathrm{c}}\delta_{ij}\delta_{nm} \\ &+\omega_{\mathrm{c}}A_0\mu(R_j)\big(\sqrt{n+1}\delta_{m,n+1} - \sqrt{n}\delta_{m,n-1}\big)\delta_{ij} + \frac{\omega_{\mathrm{c}}}{\hbar}{A_0}^2\mu_0^2(R_j)\delta_{ij}\delta_{nm},\end{aligned} \tag{78}$$

where the matrix elements of the nuclear kinetic energy operator $\langle R_i|\hat{T}_r|R_j\rangle = \langle R_i|\frac{\hat{P}^2}{2M}|R_j\rangle$ are evaluated using the Fourier grid method[18,19] as follows

$$\langle R_i|\hat{T}|R_j\rangle = \frac{\hbar^2}{2M}\cdot\frac{\pi^2}{3(\Delta R)^2}\Big(1 + \frac{2}{N^2}\Big)\delta_{ij} + \frac{\hbar^2}{2M}\cdot\frac{2(-1)^{j-i}\pi^2}{(\Delta R\cdot N\sin\big(\frac{\pi(j-i)}{N}\big))^2}(1-\delta_{ij}). \tag{79}$$

In this calculation, have used $N = 400$ nuclear grid points, and $\Delta R = 0.0125$ a.u. in the range of $R \in [-2.5, 2.5]$. To ensure a fully converged results through out the range of the coupling strength, we have used 50 Fock states for computing the above matrix elements. Diagonalizing the above matrix of $\hat{H}_{\mathrm{vpl}}$ provides the results of the polariton eigenspectrum and polariton eigenstates.

Diagonalizing to obtain the polariton eigenvalue $\mathcal{E}_\nu$, as well as the polariton eigenvector

$$|\Phi_\nu\rangle = \sum_{n,j} b^\nu_{n,j} |R_j, n\rangle, \tag{80}$$

where $\{b^\nu_{n,j}(R) = \langle R_j, n|\Phi_\nu\rangle\}$ are the eigenvectors of $\hat{H}_{\mathrm{vpl}}$.

Supplementary Fig. 4 presents the Rabi splitting calculated by directly diagonalizing $\hat{H}_{\mathrm{vpl}}$ (black dots) compared to the simple analytical expression (red lines) $\hbar\Omega_R = 2\sqrt{\frac{\hbar}{2M\omega_0}}\chi \cdot \mu'_0$ where we approximate the dipole as $\mu(\hat{R}) \approx \mu_0 + \mu'_0(\hat{R} - R_0)$, where $\mu_0 = \mu(R_0)$ and $\mu'_0 = \frac{\partial \mu(R)}{\partial R}|_{R_0}$. For $\eta < 0.1$ both the numerical and the analytical result remains close to each other and deviates for larger $\eta > 0.1$. We note that the deviation at larger η is caused by the non-linearity of the dipole moment in the vicinity $R_0 \approx -1.74$ a.u.

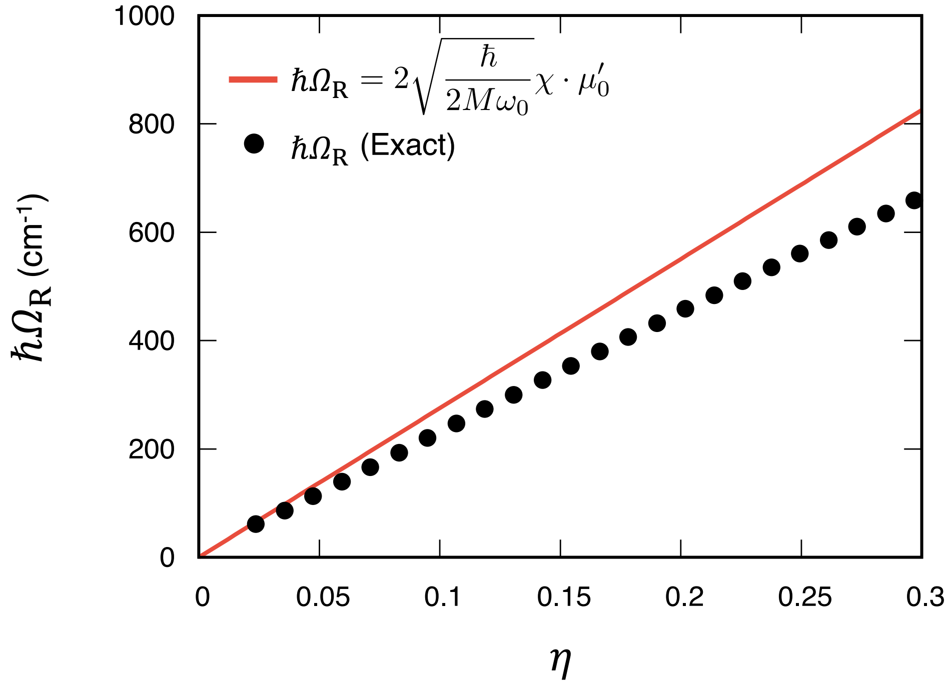

Supplementary Figure 4: Rabi splitting $\hbar\Omega_R$ induced by light-matter interactions, computed numerically exactly (black dots) compared with the analytical expression for Rabi splitting that assumes a linear dipole $\mu \approx \mu_0 + \mu'_0 R$.

To compute the absorption spectrum of the molecule-cavity hybrid system, we use a simple analytical scheme,[21] and a phenomenological width parameter $\varepsilon = 21.95$ cm^{-1} to account for the

broadening of the absorption spectrum observed in the recent experiments.[22] The absorption cross section $\sigma(\mathcal{E})$ as a function of excitation energy $\mathcal{E}$ is expressed[21,23] as follows

$$\sigma(\mathcal{E}) = \frac{4\pi\mathcal{E}}{c}\mathrm{Im}\Big[\sum_{\nu\neq 0}\frac{|\langle\Phi_\nu|\mu(\hat{R})|\Phi_0\rangle|^2}{\mathcal{E}_\nu - \mathcal{E}_0 - \mathcal{E} - i\varepsilon}\Big], \tag{81}$$

where $\mathcal{E}_\nu$ is the ν_{th} vibrational polaritonic eigenenergy obtained by solving Supplementary Eq. 77, and $\mathcal{E}_0$ is ground vibrational polaritonic eigenenergy of $\hat{H}_{\mathrm{vpl}}$, and c is the speed of the light. The transition matrix element is computed as follows

$$\langle\Phi_\nu|\mu(\hat{R})|\Phi_0\rangle = \sum_n\sum_j\langle\Phi_\nu|R_j,n\rangle\Big(R_j + \sum_i c_i^*(R_j)c_i(R_j)r_i\Big)\langle R_j,n|\Phi_0\rangle, \tag{82}$$

where $\mu(\hat{R})$ is expressed in Supplementary Eq. 75, i and j are indices for electronic grid points r_i and nuclear grid points R_j, respectively, and n is the index of the vacuum's Fock state. In addition, $c_i(R_j)$ is the expansion coefficients of the adiabatic electronic states obtained by diagonalizing $\hat{H}_{\mathrm{el}}(R_j) = \sum_{k,l}\langle r_l|\hat{H}_{\mathrm{el}}(R_j)|r_k\rangle|r_l\rangle\langle r_k|$ (matrix elements provided in Supplementary Eq. 72) at the particular nuclear configuration R_j.

c. Parameterization of the Potential Energy Surface. To facilitate the numerical simulation of the reactive recrossing dynamics, the potential energy $E(R)$ and the permanent dipole $\mu(R)$ are obtained from FGH quantum calculation are fitted to analytical functions. For $E(R)$, we used a cosine series to fit the potential energy surface (PES)

$$E(R) = a_0 + \sum_{i=1}^{\mathcal{N}} b_i\cdot\cos(c_i\cdot R). \tag{83}$$

A total number of $\mathcal{N} = 8$ cosine functions were used to represent the ground-state PES, with the corresponding parameters listed below in Supplementary Table 2.

Supplementary Table 2: Parameters of the ground-state PES $E(R)$.

a_0 (a.u.)	9.929							
b_{i} (a.u.)	-19.080	14.133	-8.670	4.441	-1.842	0.594	-0.135	0.017
c_{i} (1/a.u.)	0.448	0.896	1.344	1.792	2.240	2.688	3.316	3.580

Similarly, we fitted $\mu(R)$ with the following sine series

$$\mu(R) = \sum_{i=1}^{N} a_i \cdot \sin(b_i \cdot R + c_i), \tag{84}$$

where $\mathcal{N} = 6$ sine functions to fit the DM with the parameters detailed in Supplementary Table 84.

Supplementary Table 3: Parameters used to fit the ground-state dipole $\mu(R)$.

a_i (a.u.)	1.350	0.078	0.319	0.020	0.001	-0.005
b_i (1/a.u.)	0.802	3.292	1.957	4.722	7.745	6.217
c_i (unitless)	π	π	$-\pi$	π	π	0.000

The comparison between the numerical PES and dipole obtained from the Shin-Metiu model and the fitted ones are shown in Supplementary Fig. 5.

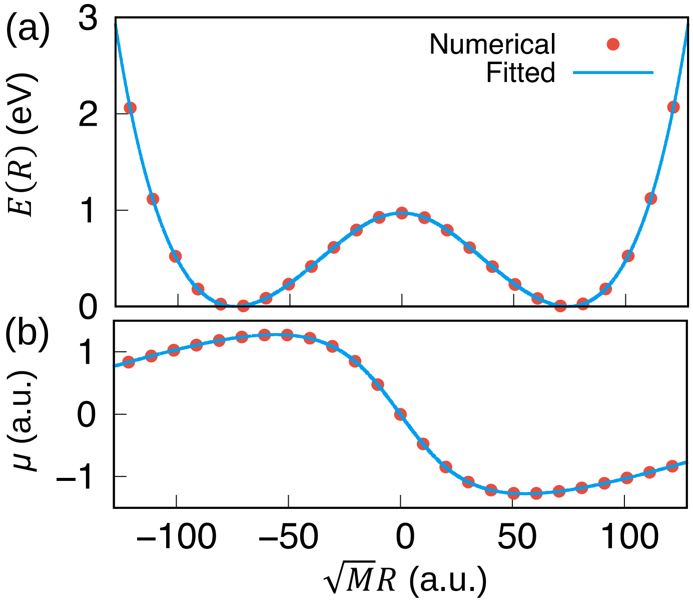

Supplementary Figure 5: Comparison between the numerical and fitted PES and DM with respect to the mass-weighted coordinate $\sqrt{M}R$.

d. Numerical Calculations of $\kappa(t)$ To compute $\kappa(t)$ from the flux-side correlation function in Eq. 4 of the main text, the photon mode q_c and the molecular reaction coordinate R was treated classically. Because the memory effect of the phonon bath has a negligible influence on κ (as shown in Supplementary Fig. 1), we use Langevin dynamics to simulate the influence of $\hat{H}_\mathrm{vib}$ on R.

The Hamilton's equations of motion in Supplementary Eq. 34-Supplementary Eq. 37 is *equivalent* to generalized Langevin equation (GLE) for the reaction coordinate R as follows

$$M\ddot{R} = -\nabla_R H_{\mathrm{pl}}(R, q_{\mathrm{c}}) - M\int_0^t d\tau \xi_{\mathrm{p}}(t-\tau)\dot{R}(\tau) + \sqrt{M}\mathcal{F}_{\mathrm{p}}(t), \tag{85}$$

where the friction kernel and the random force for the phonon bath are

$$\xi(t) = \frac{1}{M}\sum_k \frac{c_k^2}{M_k\omega_k^2}\cos(\omega_k t) = \frac{2}{\pi M}\int_0^\infty d\omega \frac{J(\omega)}{\omega}\cos(\omega t) \tag{86}$$

$$\mathcal{F}_{\mathrm{p}}(t) = -\sum_k \frac{c_k}{\sqrt{M}}\Big(R_k(0)\cos(\omega_k t) + \frac{R_k(0)}{\omega_k}\sin(\omega_k t)\Big), \tag{87}$$

according to Supplementary Eq. 52 and Supplementary Eq. 54. The derivation procedure is same as those used in deriving Supplementary Eq. 58, with the only difference that in Eq. 58 $-\nabla_R H_{\mathrm{pl}}(R, q_c)$ is not explicitly expressed in q_{c} (in Supplementary Eq. 35). In addition, $J(\omega)$ is the spectral density expressed in Supplementary Eq. 27, M and $\{M_k\}$ are the mass associated with the reaction coordinate R and the photon bath mode $\{R_k\}$, respectively. The friction and random force satisfy the fluctuation-dissipation relation $\langle \mathcal{F}_{\mathrm{p}}(0)\mathcal{F}_{\mathrm{p}}(t)\rangle = Mk_{\mathrm{B}}T\xi_{\mathrm{p}}(t)$.

For the current model systems, $J(\omega) = \zeta\omega e^{-\omega/\omega_{\mathrm{p}}}$ (Supplementary Eq. 27), we have

$$\xi_{\mathrm{p}}(t) = \frac{2}{\pi M}\int_0^\infty d\omega \frac{J(\omega)}{\omega}\cos(\omega t) = \frac{2}{\pi M}\int_0^\infty d\omega \zeta e^{-\omega/\omega_{\mathrm{p}}}\cos(\omega t) = \frac{2\zeta}{M}\frac{1}{\pi}\frac{\omega_{\mathrm{p}}}{1+(\omega_{\mathrm{p}}t)^2}. \tag{88}$$

To simplify our theoretical analysis of the transmission coefficient, We assume the Markovian approximation under the limit of $\omega_{\mathrm{p}} \to \infty$ (ω_{p} is larger than all physical frequency of the system), leading to the friction kernel as follows

$$\lim_{\omega_{\mathrm{p}}\to\infty}\xi_{\mathrm{p}}(t) = \lim_{\omega_{\mathrm{p}}\to\infty}\frac{2\zeta}{M}\frac{1}{\pi}\frac{\omega_{\mathrm{p}}}{1+(\omega_{\mathrm{p}}t)^2} = \frac{2\zeta}{M}\delta(t). \tag{89}$$

Under this limit, the and the random force correlation function becomes

$$\langle \mathcal{F}_{\mathrm{p}}(0)\mathcal{F}_{\mathrm{p}}(t)\rangle = Mk_{\mathrm{B}}T\lim_{\omega_{\mathrm{p}}\to\infty}\xi_{\mathrm{p}}(t) = 2k_{\mathrm{B}}T\zeta\delta(t), \tag{90}$$

and the memory kernel in GLE is evaluated as

$$\int_0^t d\tau \xi_{\rm p}(t-\tau)\dot{R}(\tau) = \int_0^t d\tau \xi_{\rm p}(\tau)\dot{R}(t-\tau) \approx \left[\int_0^\infty d\tau \xi_{\rm p}(\tau)\right]\dot{R}(t) = \frac{\zeta}{M}\dot{R}(t), \tag{91}$$

where the first equality is from the convolution theorem of Laplace transform, $\mathcal{L}^{-1}[\tilde{\xi}_{\rm p}\cdot\tilde{\dot{R}}] = \int_0^t d\tau\xi_{\rm p}(t-\tau)\dot{R}(\tau) = \int_0^t d\tau\xi_{\rm p}(\tau)\dot{R}(t-\tau)$.

The GLE in Supplementary Eq. 85 under the Markovian limit is thus expressed as the following Langevin equation

$$M\ddot{R} = -\nabla_R H_{\rm pl}(R, q_c) - \zeta\dot{R} + \mathcal{F}_{\rm p}(t), \tag{92}$$

where the random force $\mathcal{F}_{\rm p}(t)$ satisfies $\langle\mathcal{F}_{\rm p}(0)\mathcal{F}_{\rm p}(t)\rangle = Mk_{\rm B}T\xi_{\rm p}(t) = 2k_{\rm B}T\zeta\delta(t)$,the force acting on the nuclear reaction coordinate is $-\nabla_R H_{\rm pl}(R,q_{\rm c}) = -\sqrt{\frac{2\omega_{\rm c}}{\hbar}}\chi\big(q_{\rm c} + \sqrt{\frac{2}{\hbar\omega_{\rm c}^3}}\chi\cdot\mu(R)\big)\cdot\nabla_R\mu(R) - \nabla_R E(R)$, and the classical equation of motion for the photonic DOF is

$$\ddot{q}_{\rm c} = -\nabla_{q_{\rm c}} H_{\rm pl}(R, q_c) = -\omega_{\rm c}^2\Big(q_{\rm c} + \sqrt{\frac{2}{\hbar\omega_{\rm c}^3}}\chi\cdot\mu(R)\Big) = F_{q_{\rm c}} \tag{93}$$

To solve the above coupled equations numerically, we have used a modified velocity Verlet integrator[24,25] for the molecular DOF R and a velocity Verlet integrator for the photonic DOF $q_{\rm c}$ as follows

$$R(t+dt) = R(t) + \dot{R}(t)dt + A(t) \tag{94}$$

$$\dot{R}(t+dt) = \dot{R}(t) + \frac{dt}{2}[F_R(t+dt) + F_R(t)] - dt\gamma\dot{R}(t) + \sigma\sqrt{dt}\xi(t) - \gamma A(t) \tag{95}$$

$$\dot{q}_{\rm c}(t+\frac{dt}{2}) = \dot{q}_{\rm c}(t) + \frac{1}{2}F_{q_{\rm c}}(t)dt \tag{96}$$

$$q_{\rm c}(t+dt) = q_{\rm c}(t) + \dot{q}_{\rm c}(t)dt \tag{97}$$

$$\dot{q}_{\rm c}(t+dt) = \dot{q}_{\rm c}(t+\frac{dt}{2}) + \frac{1}{2}F_{q_{\rm c}}(t+dt)dt, \tag{98}$$

where the function $A(t)$ is[24,25]

$$A(t) = \frac{1}{2}dt^2(F_{\rm R}(t) - \gamma\dot{R}(t)) + \sigma dt^{3/2}\left(\frac{1}{2}\xi(t) + \frac{1}{2\sqrt{3}}\theta(t)\right), \tag{99}$$

and $\xi(t)$ and $\theta(t)$ are Gaussian random numbers generated at time t.

Alternatively, we also directly simulate the full Hamiltonian $H(R, q_c, \{R_k\})$ in Supplementary Eq. 29 with the explicit time-dependent propagation of the phonon bath $\{R_k\}$. A simple velocity Verlet algorithm is used to solving the following equations of motion

$$\dot{R} = \frac{\partial H}{\partial P} = p, \quad \dot{R}_k = \frac{\partial H}{\partial P_k} = P_k, \quad \dot{q}_c = \frac{\partial H}{\partial p_c} = p_c, \tag{100}$$

$$\dot{P} = -\frac{\partial H}{\partial R} = -\frac{\partial E(R)}{\partial R} - \sqrt{\frac{2\omega_c}{\hbar}}\chi\Big(q_c + \sqrt{\frac{2}{\hbar\omega_c^3}}\chi\cdot\mu(R)\Big)\cdot\frac{\partial\mu(R)}{\partial R} - \sum_k c_k\Big(R_k + \frac{c_k}{M_k\omega_k^2}R\Big), \tag{101}$$

$$\dot{P}_k = -\frac{\partial H}{\partial R_k} = -M_k\omega_k^2\Big(R_k + \frac{c_k}{M_k\omega_k^2}R\Big) \tag{102}$$

$$\dot{p}_c = -\frac{\partial H}{\partial q_c} = -\omega_c^2\Big(q_c + \sqrt{\frac{2}{\hbar\omega_c^3}}\chi\cdot\mu(R)\Big). \tag{103}$$

The transmission coefficient is numerically calculated from the flux-side correlation function formalism[26–28] as follows

$$\kappa(t) = \frac{\langle\mathcal{F}(0)\cdot h[R(t) - R_\ddagger]\rangle}{\langle\mathcal{F}(0)\cdot h[\dot{R}_\ddagger(0)]\rangle}, \tag{104}$$

where $h[R - R_\ddagger]$ is the Heaviside function of the reaction coordinate R, with the dividing surface $R_\ddagger$ that separate the reactant and the product regions (for the model system studied here, $R_\ddagger = 0$), the flux function $\mathcal{F}(t) = \dot{h}(t) = \delta[R(t) - R_\ddagger]\cdot\dot{R}(t)$ measures the reactive flux across the dividing surface (with $\delta(R)$ as the Dirac delta function), and $\langle ...\rangle$ represents the canonical ensemble average (subject to constrain on the dividing surface which is enforced by $\delta[R(t) - R_\ddagger]$ inside $\mathcal{F}(t)$). Further, $\dot{R}_\ddagger(0)$ represents the initial velocity of the nuclei on the dividing surface. The above flux-side formalism of the reaction rate can be derived from Onsager's regression hypothesis, with derivations presented in standard text books (e.g., Ref. 28).

All simulations were performed under $T = 300$ K. The time step dt we chose was 4 a.u., which was carefully checked to produce stable integration for all simulations. From a constraint MD trajectory with $R_\ddagger = 0$, the constrained configurations $\{q_c, R_\ddagger\}$ (for the Langevin dynamics) or $\{q_c, R_\ddagger, \{R_k\}\}$ (for the explicit bath) are sampled for every 270 fs along the constrained trajectory. A total of 100,000 configurations are released from the dividing surface, with the initial velocities randomly sampled from the classical Maxwell-Boltzmann distribution. Each trajectory

is propagated for 200 fs, which guaranteed that the flux-side correlation function would plateau.

For the Langevin dynamics simulation of the bath, the equations of motion in Supplementary Eq. 92 and Supplementary Eq. 93 are numerically propagated using the algorithm in Supplementary Eq. 95-98. The friction parameter was chosen to be $\zeta = 49.6$ meV, according to the spectral density of the $\hat{H}_{\mathrm{vib}}$. For the explicit bath propagation, the equations of motion in Supplementary Eq. 92 and Supplementary Eq. 93 are propagated with velocity Verlet. The numerical results are presented as dots in Supplementary Fig. 1b. When simulating the model system with a high frequency phonon bath $\omega_{\mathrm{p}} = 5\omega_0$ (green dots in Supplementary Fig. 1b), we have reduced the time step in velocity Verlet with $dt = 1$ a.u. The direct numerical simulations from Supplementary Eq. 104 agree well with the values predicted from the κ_{GH} when explicitly considering the phonon bath as non-Markovian environment (by solving Supplementary Eq. 61). Moreover, explicitly treating the phonon bath as non-Markovian environment (blue curve in Supplementary Fig. 1b) generates essentially the same results compared to treating the phonon bath through Langevin dynamics (red curve in Supplementary Fig. 1b).

Supplementary Note 6. Additional Numerical Results

Supplementary Fig. 6 presents the representative reactive trajectories on the cavity BO potential energy surfaces, with $\eta = 0.188$. Panel (a) presents the transmission coefficient κ (when $t \to t_{\mathrm{p}}$) as a function of the photon frequency ω_{c} with three normalized coupling constant η. The results are obtained from the GH theory (solid line) as well as the direct numerical simulation of Eq. 104 (filled circles).

In panels b-d, with the black solid lines, indicate representative trajectories. At a very low frequency, $\hbar\omega_{\mathrm{c}} = 1$ meV as shown in panel b, the photon coordinate essentially remains frozen compared to the dynamics of the reaction coordinate R during the course of the reaction. As a result, under this frozen solvent limit, the transmission coefficient remains close to the no-coupling scenario. At $\hbar\omega_{\mathrm{c}} = 25$ meV in panel c, with $\frac{|\mathcal{C}_{\ddagger}|}{\omega_{\mathrm{c}}} \gg \omega_{\mathrm{b}}$, the light-matter interactions leads to the dynamical caging of the reaction coordinate at the barrier top leading to a significant decrease in the transmission coefficient κ_{GH}. When the photon frequency is further increased, the reactant and the product wells become separated with a narrow channel as shown in panel d when $\hbar\omega_{\mathrm{c}} = 1$

eV. At such a high photon frequency, the channel connecting the reactant becomes extremely narrow[29] (much narrower than the usual dynamical caging scenario depicted in panel c, such that the reactive trajectories almost follow a straight path and is no longer caged near the dividing surface. As opposed to the dynamic caging regime, the transmission coefficient in panel d is less suppressed than the minimum κ.

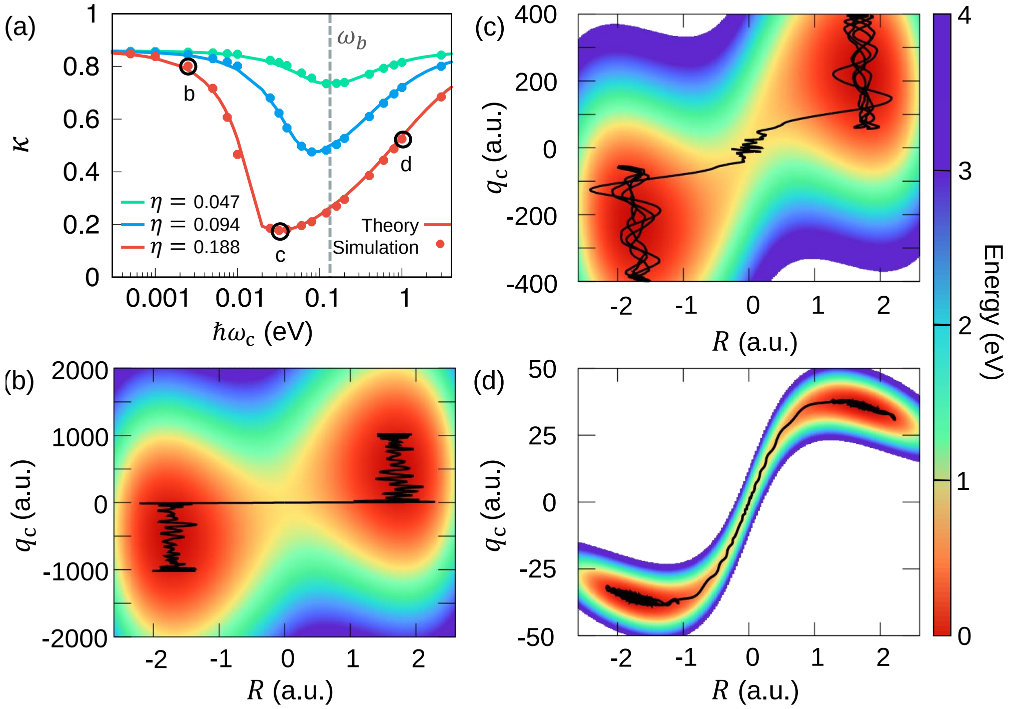

Supplementary Figure 6: Resonance effect of the transmission coefficient κ by changing the photon frequency $\hbar\omega_c$. (a) Transmission coefficient κ as a function of the photon frequency color coded to represent different coupling strength η. Cavity Born-Oppenheimer surfaces as a function of q_c and R at $\eta = 0.188$ (corresponding to the blue solid line in panel (a)) and at (b) $\omega_c = 1$ meV, (c) 25 meV, and (d) 1.0 eV with the black solid line indicating a representative reactive trajectory.

Supplementary Fig. 7 presents the time-dependent transmission coefficient with $\eta = 0.188$ when treating the phonon bath $\hat{H}_{\text{vib}}$ with Langevin dynamics. The time-dependent $\kappa(t)$ at different ω_c values are presented, and the resonant behavior is also clearly indicated by the long-time plateau value of $\kappa(t)$, which decrease as ω_c, then increase as the further increase of ω_c.

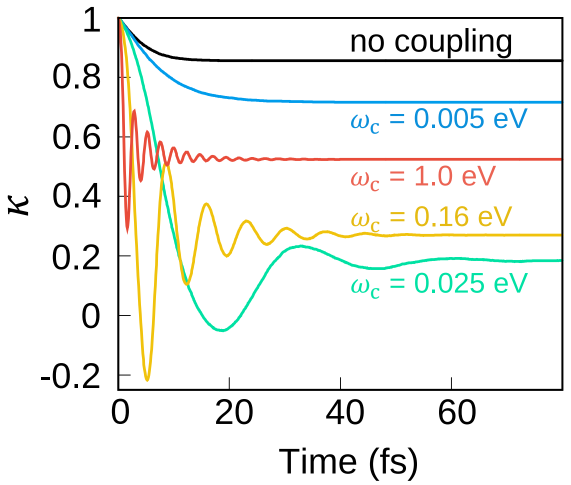

Supplementary Figure 7: Transmission coefficient $\kappa(t)$ at various photon frequencies ω_c. The light-matter coupling η is fixed at $\eta = 0.188$ for all simulations.

References

(1) Power, E. A.; Zienau, S. Coulomb gauge in non-relativistic quantum electro-dynamics and the shape of spectral lines. *Philos. Trans. R. Soc. London, Ser. A* **1959**, *251*, 427–454.

(2) Cohen-Tannoudji, C.; Dupont-Roc, J.; Grynberg, G. *Photons and Atoms: Introduction to Quantum Electrodynamics*; John Wiley & Sons, Inc.: Hoboken, 1989.

(3) Stefano, O. D.; Settineri, A.; Macri, V.; Garziano, L.; Stassi, R.; Savasta, S.; Nori, F. Resolution of gauge ambiguities in ultrastrong-coupling cavity quantum electrodynamics. *Nature Phys.* **2019**, *15*, 803–808.

(4) Göppert-Mayer, M. Elementary Processes With Two Quantum Transitions. *Ann. Phys. (Berlin)* **2009**, *18*, 466–479.

(5) Flick, J.; Ruggenthaler, M.; Appel, H.; Rubio, A. Atoms and Molecules in Cavities, from Weak to Strong Coupling in Quantum-Electrodynamics (QED) Chemistry. *Proc. Natl. Acad. Sci. U. S. A.* **2017**, *114*, 3026.

(6) Rokaj, V.; Welakuh, D. M.; Ruggenthaler, M.; Rubio, A. Light–matter interaction in the

longwavelength limit: no ground-state without dipole self-energy. *J. Phys. B: At. Mol. Opt. Phys.* **2018**, *51*, 034005.

(7) Schäfer, C.; Ruggenthaler, M.; Rubio, A. Ab initio nonrelativistic quantum electrodynamics: Bridging quantum chemistry and quantum optics from weak to strong coupling. *Phys. Rev. A* **2018**, *98*, 043801.

(8) Hoffmann, N. M.; Schäfer, C.; Säkkinen, N.; Rubio, A.; Appel, H.; Kelly, A. Benchmarking semiclassical and perturbative methods for real-time simulations of cavity-bound emission and interference. *J. Chem. Phys.* **2019**, *151*, 244113.

(9) Li, T. E.; Chen, H.-T.; Nitzan, A.; Subotnik, J. E. Quasiclassical modeling of cavity quantum electrodynamics. *Phys. Rev. A* **2020**, *101*, 033831.

(10) Campos-Gonzalez-Angulo, J. A.; Yuen-Zhou, J. Polaritonic normal modes in transition state theory. *J. Chem. Phys.* **2020**, *152*, 161101.

(11) Shin, S.; Metiu, H. Nonadiabatic Effects on the Charge Transfer Rate Constant: A Numerical Study of a Simple Model System. *J. Chem. Phys.* **1995**, *102*, 9285–9295.

(12) Caldeira, A. O.; Leggett, A. J. Influence of Dissipation on Quantum Tunneling in Macroscopic Systems. *Phys. Rev. Lett.* **1981**, *46*, 211–214.

(13) Makri, N. The Linear Response Approximation and its Lowest Order Corrections: An Influence Functional Approach. *J. Phys. Chem. B* **1999**, *103*, 2823–2829.

(14) Nitzan, A. *Chemical Dynamics in Condensed Phases: Relaxation, Transfer and Reactions in Condensed Molecular Systems*; Oxford Univ. Press: Oxford, 2006.

(15) Zwanzig, R. *Nonequilibrium Statistical Mechanics*; Oxford Univ. Press: Oxford, 2001.

(16) Henriksen, N. E.; Hansen, F. Y. *Theories of molecular reaction dynamics: the microscopic foundation of chemical kinetics*; Oxford Univ. Press: Oxford, 2008.

(17) Pollak, E. Theory of activated rate processes: A new derivation of Kramers' expression. *J. Chem. Phys.* **1986**, *85*, 865–867.

(18) Marston, C. C.; Balint-Kurti, G. G. The Fourier grid Hamiltonian method for bound state eigenvalues and eigenfunctions. *J. Chem. Phys.* **1989**, *91*, 3571–3576.

(19) Tannor, D. *Introduction to Quantum Mechanics: A Time-Dependent Perspective*; Univ. Science Books: Sausalito, 2007.

(20) Feist, J.; Galego, J.; Garcia-Vidal, F. J. Polaritonic Chemistry with Organic Molecules. *ACS Photonics* **2018**, *5*, 205–216.

(21) Galego, J.; Garcia-Vidal, F. J.; Feist, J. Cavity-Induced Modifications of Molecular Structure in the Strong-Coupling Regime. *Phys. Rev. X* **2015**, *5*, 041022.

(22) Thomas, A.; Lethuillier-Karl, L.; Nagarajan, K.; Vergauwe, R. M. A.; George, J.; Chervy, T.; Shalabney, A.; Devaux, E.; Genet, C.; Moran, J.; Ebbesen, T. W. Tilting a ground-state reactivity landscape by vibrational strong coupling. *Science* **2019**, *363*, 615–619.

(23) Rescigno, T. N.; McKoy, V. Rigorous method for computing photoabsorption cross sections from a basis-set expansion. *Phys. Rev. A* **1975**, *12*, 522–525.

(24) Tuckerman, M. *Statistical Mechanics: Theory and Molecular Simulation*; Oxford Univ. Press: Oxford, 2010.

(25) Vanden-Eijnden, E.; Ciccotti, G. Second-order integrators for Langevin equations with holonomic constraints. *Chemical Physics Letters* **2006**, *429*, 310 – 316.

(26) Frenkel, D.; Smit, B. *Understanding Molecular Simulation*; Elsevier: San Diego, 2002.

(27) Miller, W. H.; Schwartz, S. D.; Tromp, J. W. Quantum Mechanical Rate Constants for Bimolecular Reactions. *J. Chem. Phys.* **1983**, *79*, 4889–4898.

(28) Chandler, D.; Wu, D. *Introduction to Modern Statistical Mechanics*; Oxford Univ. Press: Oxford, 1987.

(29) Truhlar, D. G.; Garrett, B. C. Multidimensional Transition State Theory and the Validity of Grote-Hynes Theory. *J. Phys. Chem. B* **2000**, *104*, 1069–1072.
